# Synthesis, Biological Evaluation and Molecular Modeling of Substituted Indeno[1,2-*b*]indoles as Inhibitors of Human Protein Kinase CK2

**DOI:** 10.3390/ph8020279

**Published:** 2015-06-08

**Authors:** Faten Alchab, Laurent Ettouati, Zouhair Bouaziz, Andre Bollacke, Jean-Guy Delcros, Christoph G.W. Gertzen, Holger Gohlke, Noël Pinaud, Mathieu Marchivie, Jean Guillon, Bernard Fenet, Joachim Jose, Marc Le Borgne

**Affiliations:** 1 EA 4446 Biomolécules Cancer et Chimiorésistances, SFR Santé Lyon-Est CNRS UMS3453 - INSERM US7, Faculté de Pharmacie - ISPB, Université Lyon 1, 8 avenue Rockefeller, F-69373, Lyon Cedex 8, France; E-Mails: fatenalchab@yahoo.com (F.A.); laurent.ettouati@univ-lyon1.fr (L.E.); zouhair.bouaziz@univ-lyon1.fr (Z.B.); 2 Institut für Pharmazeutische und Medizinische Chemie, PharmaCampus, Westfälische Wilhelms-Universität Münster, Corrensstr. 48, 48149 Münster, Germany; E-Mails: andre.bo@uni-muenster.de (A.B.); joachim.jose@uni-muenster.de (J.J.); 3 Laboratoire Récepteurs à dépendance, UMR INSERM U1052/CNRS 5286, Centre de Recherche en Cancérologie de Lyon, Centre Léon Bérard, Cheney A, 28 rue Laënnec, F-69008, Lyon, France; E-Mail: jean-guy.delcros@lyon.unicancer.fr; 4 Institut für Pharmazeutische und Medizinische Chemie, Heinrich-Heine-Universität Düsseldorf, Universitätsstr. 1, 40225 Düsseldorf, Germany; E-Mails: christoph.gertzen@uni-duesseldorf.de (C.G.W.G.); gohlke@uni-duesseldorf.de (H.G.); 5 ISM - CNRS UMR 5255, Université de Bordeaux, 351 cours de la Libération, F-33405 Talence cedex, France; E-Mail: noel.pinaud@u-bordeaux.fr; 6 ICMCB, UPR 9048, CNRS, Université de Bordeaux, 87, avenue du Docteur Schweitzer, F-33600 Pessac, France; E-Mail: mathieu.marchivie@u-bordeaux.fr; 7 Laboratoire ARNA, INSERM U869, UFR des Sciences Pharmaceutiques, Université de Bordeaux, 146 rue Léo Saignat, F-33076 Bordeaux cedex, France; E-Mail: jean.guillon@u-bordeaux.fr; 8 Centre Commun de RMN, Université de Lyon, F-69003 Lyon, France; E-Mail: bernard.fenet@univ-lyon1.fr; 9 ESCPE Lyon, Université Lyon 1, 43 Bd du 11 Novembre 1918, F-69616 Villeurbanne Cedex, France

**Keywords:** indeno[1,2-*b*]indoles, synthesis, protein kinase CK2, inhibitory activity, molecular modeling, cytotoxicity

## Abstract

Due to their system of annulated 6-5-5-6-membered rings, indenoindoles have sparked great interest for the design of ATP-competitive inhibitors of human CK2. In the present study, we prepared twenty-one indeno[1,2-*b*]indole derivatives, all of which were tested *in vitro* on human CK2. The indenoindolones **5a** and **5b** inhibited human CK2 with an IC_50_ of 0.17 and 0.61 µM, respectively. The indeno[1,2-*b*]indoloquinone **7a** also showed inhibitory activity on CK2 at a submicromolar range (IC_50_ = 0.43 µM). Additionally, a large number of indenoindole derivatives was evaluated for their cytotoxic activities against the cell lines 3T3, WI-38, HEK293T and MEF.

## 1. Introduction

**Figure 1:**
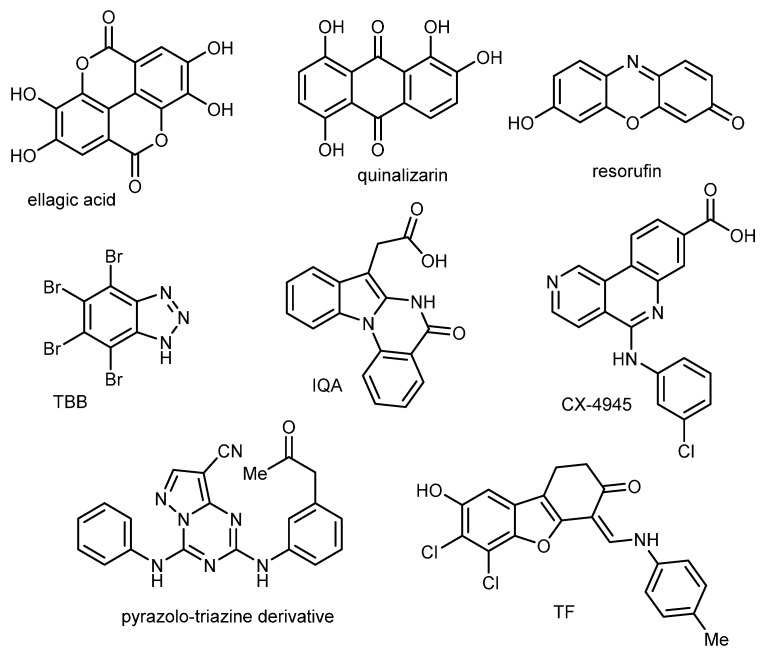
Examples of known CK2 inhibitors.

Protein kinase CK2 is a ubiquitous serine/threonine kinase found in all eukaryotic cells. Known since 1954 [1], CK2 is a newly validated therapeutic target (e.g., pancreatic adenocarcinoma, breast and prostate carcinoma, hematopoietic tumors) [2,3,4,5], is ideally suited for drug design [6,7], and will be a major target for inhibition for the next decades [8]. Actually, numerous ATP-competitive inhibitors have been identified [9,10,11], which consist of small and planar heterocyclic scaffolds, able to fit into the nucleotide-binding pocket of CK2α and to displace the ATP. Among these inhibitors, are (i) polyhalogenated benzimidazole and benzotriazole derivatives, (ii) flavonoids, ellagic acid and coumarins, (iii) anthraquinone, xanthenone and fluorenone, (iv) pyrazolo-triazine derivatives, (v) carboxyl acid derivatives and, (vi) benzofuran derivatives (Figure 1).

**Figure 2:**
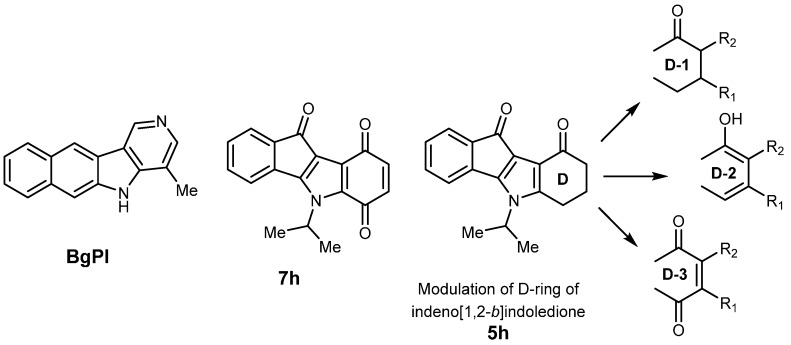
Scaffolds for designing CK2 inhibitors and D-ring modulation on indeno[1,2-*b*]indoledione.

Recently, some new CK2 inhibitors were designed using tricyclic and tetracyclic scaffolds [12,13]. Benzo[*g*]pyridoindole (BgPI) and indeno[1,2-*b*]indole scaffolds have sparked great interest for the design of ATP-competitive inhibitors of CK2 (Figure 2). For example, 5-isopropyl-5*H*-indeno[1,2-*b*]indole-6,9,10-trione **7h** inhibits CK2 with an IC_50_ value of 5.05 µM [14]. Furthermore, this *N*-isopropyl derivative **7h** inhibited the growth of a series of different tumor cell lines (5637, SISO, KYSE-70, MCF-7, LCLC, A427) [14]. Indenoindoles represent a wide class of synthetic compounds through the system of annulated 6-5-5-6-membered rings. They are of great interest due to their potential for wide biological applications, especially for drug development in oncology (e.g., DNA and topoisomerase-II, CK2) [15,16].

Structure-Activity Relationship (SAR) studies were ongoing to evaluate the influence of the nature and position of substituents on the four rings A-D. The first modifications on the C-ring showed that introduction of a non-bulky group (e.g., ethyl, isopropyl) had a favorable inhibitory effect on human CK2 [17]. Thus, we continued to explore further structural modifications on the D-ring of the indeno[1,2-*b*]indole scaffold, as described in this study (Figure 2).

## 2. Results and Discussion

**Scheme 1:**
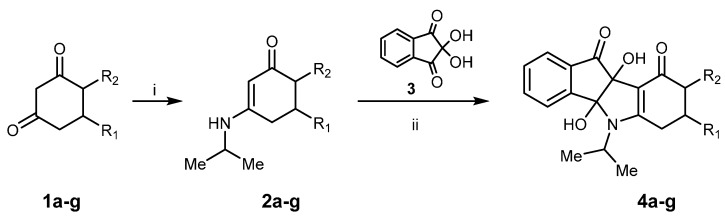
Synthesis of dihydroxyindeno[1,2-*b*]indole derivatives **4a**–**g**.

**Figure 3:**
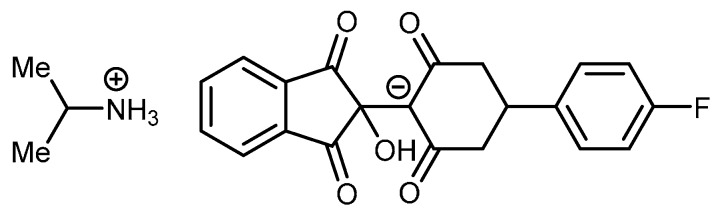
Structure of the ammonium salt **4d’**.

**Figure 4:**
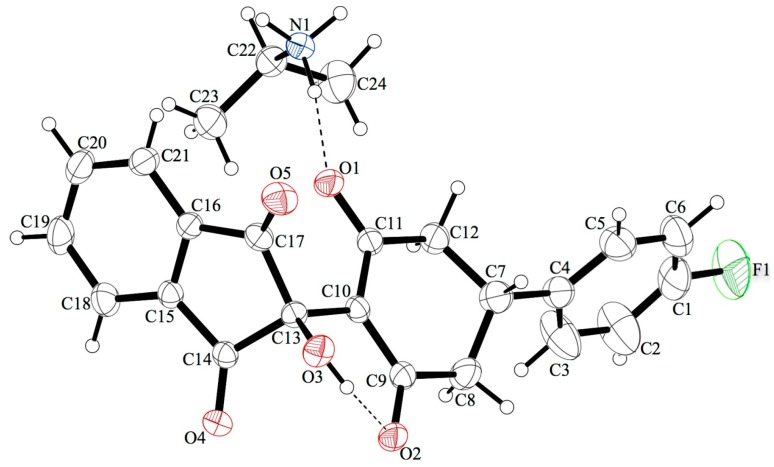
Crystal structure of **4d’** with our numbering scheme; displacement ellipsoids are drawn at the 30% probability level. Drawing was performed using the OLEX2 graphical interface [21].

Substituted cyclohexane-1,3-diones **1** were mainly commercially available except for 4-methylcyclohexane-1,3-dione **1f** and 4-isopropylcyclohexane-1,3-dione **1g**, which were synthetized in good yields by a regioselective and consecutive Michael-Claisen process starting from substituted acetone and α,β-unsaturated esters in the presence of sodium hydride at 0 °C [18]. Enaminones **2a**–**g** were prepared in good yields by nucleophilic addition of isopropylamine on substituted cyclohexane-1,3-diones **1a**–**g** followed by dehydration in toluene at reflux with a Dean-Stark water trap for 6 h (Scheme 1). Condensation of ninhydrin **3** with 3-isopropylamino-cyclohexane-2-enones **2a**–**g** at room temperature gave dihydroxyindeno[1,2-*b*]indoles **4a**–**g** (Scheme 1) in good to quantitative yields [19]. For example, dihydroxyindeno[1,2-*b*]indole **4a** (R_1_ = CH_3_, R_2_ = H) was isolated as a yellow powder in a 95% yield. In the case of compound **4d** (R_1_ = 4-fluorophenyl, R_2_ = H), two products were isolated during the work-up. The main product was identified as dihydroxyindeno[1,2-*b*]indole **4d**. The by-product was isolated after the first time round and found to be an ammonium salt of 2-[4-(4-fluorophenyl)-2,6-dioxo-cyclohexyl]-2-hydroxy-indane-1,3-dione **4d’** (Figure 3). Because formation of **4d’** was unexpected, its structure was consolidated by single-crystal X-ray diffraction analysis (Figure 4). This crystallographic study confirmed the structure in the solid state as anticipated on the basis of IR and NMR data.

*Reagents and conditions*: (i). *i*-PrNH_2_ (1 eq.), toluene, reflux, 6 h, 74%–100%. (ii). MeOH, room temp., 22 h, 46%–100%.

The key bond lengths and angles of the 2-hydroxyindane-1,3-dione moiety of **4d’** are very similar to those given in the literature for similarly substituted 2-hydroxyindane-1,3-dione derivatives [20]. The hydroxyindane-1,3-dione system (C13-C14-C15-C16-C17-C18-C19-C20-C21) of compound **4d’** is nearly planar with a mean out-of-plane deviation of 0.076 Å with the largest deviation of 0.173(2) Å for atom C13. In this compound, the cyclohexane moiety (C7-C8-C9-C10-C11-C12) is almost planar with a maximum deviation from planarity of 0.278(2) Å found for C7. **4d’** contains a cation and an anion in the solid state. The cation is formed by the ammonium function on the isopropylamine molecule. The anion is formed after hydrolysis of the intermediate imine; according to the C-O and C-C bond lengths (ranging from 1.252(3) to 1.255(3) Å, and from 1.399(3) to 1.408(3) Å, respectively) the negative charge is delocalized over C10 and the two adjacent carbonyl functions. In the crystal, the molecules are linked together by intermolecular N—H···O hydrogen bonding between the carbonyl groups of the cyclohexane-dione system and the ammonium group of the isopropylamine. An intramolecular O—H···O hydrogen bond between the hydroxyl group and the carbonyl oxygen of the cyclohexane-dione was also noticed in the solid-state conformation.

To optimize the reaction conditions, **4d** was only isolated from the mother liquor and used without further purification for the next step.

**Scheme 2:**
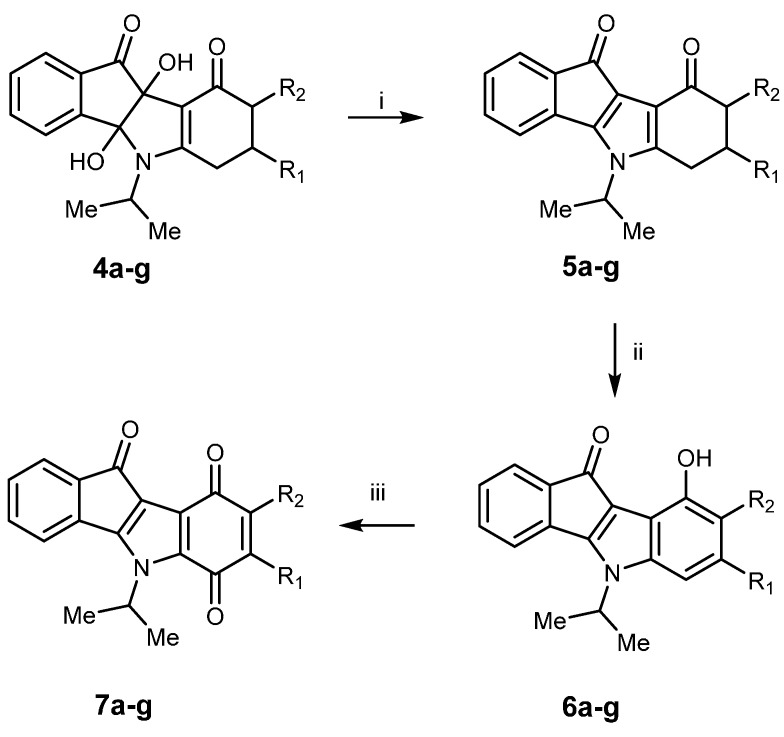
Synthesis of indeno[1,2-*b*]indole derivatives **5a**–**g**, **6a**–**g** and **7a**–**g**.

Dihydroxyindeno[1,2-*b*]indole derivatives **4a**–**g** were then converted in good yield (75% to quantitative) into 5,6,7,8-tetrahydro-indeno[1,2-*b*]indole-9,10-diones **5a**–**g** (Scheme 2) by means of *N*,*N*,*N′*,*N′*-tetraisopropylthionylamide (TIPTA) or *N*,*N*,*N′*,*N′*-tetraethylthionylamide (TETA) [19]. For example, the title compound **5a** was synthesized in a quantitative yield using TIPTA as reagent. 9-Hydroxy-5*H*-indeno[1,2-*b*]indol-10-ones **6a**–**g** were then prepared by oxidation of 5,6,7,8-tetrahydroindeno[1,2-*b*]indole-9,10-diones **5a**–**g** with Pd/C 10% at reflux in diphenyl ether. Subsequent oxidation with molecular dioxygen in the presence of salcomine, also known as ethylenebis(salicylimine) cobalt(II) salt, at room temperature afforded finally 5*H*-indeno[1,2-*b*]indole-6,9,10-triones **7a**–**g**. Yields were generally good, except for the paraquinone derivative **7g** (R_1_ = H, R_2_ = *i*-C_3_H_7_) (yield = 14%). Structural proofs for compounds **5a**–**g**, **6a**–**g** and **7a**–**g** were obtained from spectroscopic data (^1^H-NMR, ^13^C-NMR, IR, HRMS). However, owing to poor nuclear relaxation, several carbons were not assigned in the ^13^C-NMR spectra of **7b** and **7e**,**g**. Nevertheless, a previous work [22] performed to study ^1^H and ^13^C-NMR assignments of indeno[1,2-*b*]indole-10-one derivatives allowed us to achieve a complete spectral assignment for this scaffold.

In summary, three sub-series of compounds, namely D-ring mono-keto derivatives **5a**–**g**, D-ring hydroxyl derivatives **6a**–**g** and D-ring paraquinone derivatives **7a**–**g** were synthesized, purified and characterized by spectral techniques.

*Reagents and conditions*: (i). TIPTA or TETA (1.5 eq.), DMF, AcOH, room temp., 23 h, 75%–100%. (ii). Pd/C, diphenyl ether, reflux, 3 h, 20%–89%. (iii). salcomine (1.8 eq.), DMF, O_2_, room temp., 27 h, 14%–95%.

For testing the inhibitors, the recently established capillary electrophoresis CK2 activity assay was used [23]. Eleven compounds exhibited percent inhibition higher than 50% at 10 µM; corresponding IC_50_ values were calculated from the resulting dose-response curves. In the first sub-series, three compounds **5a** (R_1_ = CH_3_, R_2_ = H), **5b** (R_1_ = *i*-C_3_H_7_, R_2_ = H) and **5f** (R_1_ = H, R_2_ = CH_3_) showed a percent inhibition superior to 50%. Compound **5a** has an IC_50_ value of 0.17 µM, lower than that obtained with emodin (IC_50_ value of 0.58 µM). Still, ellagic acid and TBB remain better CK2 inhibitors than compound **5a**. After the aromatization of the D-ring, a second sub-series of compounds was studied. Compounds **6a** and **6b**, analogues of **5a** and **5b**, respectively, are active against CK2 with IC_50_ values of 1.27 and 1.45 µM, respectively. Compounds **6d** and **6e**, possessing 4-fluorophenyl or 2′-furanyl groups at position 7, displayed IC_50_ values in the micromolar range (2.77 and 3.63 µM, respectively). In the last sub-series of D-ring paraquinone derivatives **7a**–**g**, compounds with substituents CH_3_ and 2′-furanyl on position 7, are linked to CK2 inhibition, with IC_50_ values of 0.43 and 1.65 µM, respectively. Among the six regioisomers of **5a**,**b**, **6a**,**b** and **7a**,**b** (R_2_ = CH_3_, *i*-C_3_H_7_), only compound **7f** (R_2_ = CH_3_) shows a weak inhibitory activity against CK2 (IC_50_ = 4.90 µM).

After taking into account the results in our pharmacomodulation works, the introduction of a CH_3_ group at position 7 (D-ring) allowed us to identify compounds **5a**, **6a**, and **7a** as potent CK2 inhibitors. Furthermore, a second substituent, the *i*-C_3_H_7_ group at position 7, showed a favorable effect on CK2 inhibition (e.g., compounds **5b** and **6b**).

**Figure 5:**
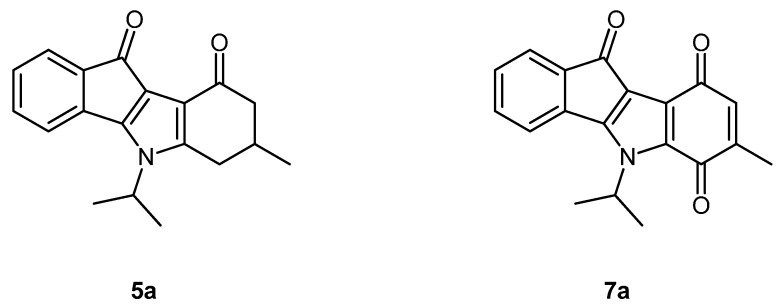
The two most potent CK2 inhibitors **5a** and **7a** of the novel indeno[1,2-*b*]indole derivatives.

The assumption that indeno[1,2-*b*]indoles as small and planar molecules exhibit their inhibitory effect towards CK2 by interacting with the ATP binding pocket had already been verified in a previous investigation [13]. In order to validate the ATP competitive mode of inhibition for the CK2 inhibitors described here, the IC_50_ values of the two most potent inhibitors, **5a** and **7a** (Figure 5), were determined at six different ATP concentrations ranging from 6 to 600 µM. IC_50_ values recorded for the two inhibitors at different ATP concentrations (0.044, 0.097, 0.17, 0.44, 0.76 and 1.17 µM for **5a** and 0.13, 0.34, 0.43, 1.27, 3.32 and 5.16 µM for **7a**) were observed to linearly increase with the concentration of ATP, thus indicating the ATP competitive mode of CK2 inhibition.

**Table 1:** Synthesized indeno[1,2-*b*]indole derivatives and inhibition of human CK2 holoenzyme.

Compound	R_1_	R_2_	Inhibition (%) ^1)^	IC_50_ ± SD (µM)
**5a**	CH_3_	H	94	0.17 ± 0.03
**5b**	*i*-C_3_H_7_	H	94	0.61 ± 0.03
**5c**	C_6_H_5_	H	7	n.d. ^2)^
**5d**	4-F-C_6_H_4_	H	12	n.d.
**5e**	2′-furanyl	H	35	n.d.
**5f**	H	CH_3_	52	9.18 ± 0.92
**5g**	H	*i*-C_3_H_7_	21	n.d.
**6a**	CH_3_	H	64	1.27 ± 0.27
**6b**	*i*-C_3_H_7_	H	60	1.45 ± 0.32
**6c**	C_6_H_5_	H	42	n.d.
**6d**	4-F-C_6_H_4_	H	59	2.77 ± 0.35
**6e**	2′-furanyl	H	65	3.63 ± 0.34
**6f**	H	CH_3_	45	n.d.
**6g**	H	*i*-C_3_H_7_	35	n.d.
**7a**	CH_3_	H	87	0.43 ± 0.12
**7b**	*i*-C_3_H_7_	H	60	4.76 ± 0.29
**7c**	C_6_H_5_	H	41	n.d.
**7d**	4-F-C_6_H_4_	H	42	n.d.
**7e**	2′-furanyl	H	82	1.65 ± 0.14
**7f**	H	CH_3_	72	4.90 ± 0.54
**7g**	H	*i*-C_3_H_7_	45	n.d.
ellagic acid	*-*	-	95	0.040 ± 0.007
emodin	*-*	-	99	0.58 ± 0.05
TBB	-	-	99	0.060 ± 0.005

^1)^ Average percent inhibition at 10 µM. ^2)^ n.d.: not determined.

**Figure 6:**
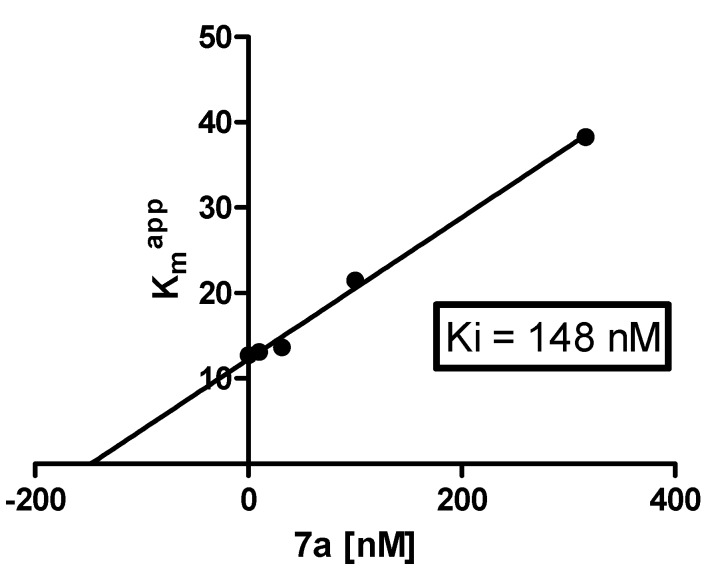
Determination of the inhibition constants for the indeno[1,2-*b*]indoloquinone **7a**. $K_{m}^{app}$were plotted against the corresponding inhibitor concentrations.

The obtained IC_50_ value at the highest ATP concentration of 600 µM was 27-fold higher for **5a** and 40-fold higher for **7a** when compared to the IC_50_ values obtained at the lowest ATP concentration of 6 µM. The IC_50_ values obtained at the different ATP concentrations were further used to determine the K_i_ values of **5a** and **7a**. For this purpose, reaction rates at different inhibitor concentrations were plotted in a Lineweaver-Burk diagram against the varying ATP concentration, resulting in $-1/K_{m}^{app}$ as given by the intercepts with the abscissa in both graphs. For determining the K_i_ values, $K_{m}^{app}$ were plotted against the different inhibitor concentrations as shown for **7a** in Figure 6. The K_i_ value with negative sign was obtained by the intercept with the abscissa and was found to be 144 ± 22 nM with a regression coefficient of R^2^ = 0.9941. For **5a**, IC_50_ values obtained with the highest ATP concentrations at 300 and 600 µM appeared to be not in the linear range in the plot, what precluded the determination of the corresponding K_i_ value. These IC_50_ values were therefore excluded for this compound. Although the resulting plot exhibited a poor regression coefficient of R^2^ = 0.8253, the K_i_ value for **5a** could be obtained by this plot and was found to be 27 ± 12 nM.

Four cell lines of various origins were used to evaluate the cytotoxicity of our target compounds **5**–**7**. The selected cell lines were (i) NIH-3T3, a cell line originally established from the primary mouse embryonic fibroblast cells, (ii) MEF, primary mouse embryonic fibroblasts (used at their early passages) prepared as previously described [24], (iii) WI-38, a human diploid cell line derived from normal embryonic lung tissue, and (iv) HEK293T, a transformed variant of the human embryonic kidney cell line HEK293. After 96 h, cytotoxicity was evaluated using the WST-1 assay. For each compound tested, the EC_50_ (effective concentration of drug needed to inhibit cell growth/viability by 50%) was generated from the dose-response curves for each cell line. Seventeen compounds were evaluated on the cell lines and then compared to TBB used as a standard. Among the three best CK2 inhibitors (**5a**, **5b**, **7a**), compounds **5a** and **5b** displayed no marked cytotoxic activity, except for compound **5b** on the MEF cell line (EC_50_ = 4.4 µM). After 96 h their EC_50_ values were superior to 10 µM, similar to TBB. No data were obtained with compound **7a** due to the lack of solubility. Four compounds, belonging to the sub-series of D-ring paraquinone derivatives (**7c**, **7d**, **7f**, **7g**), displayed cytotoxic activity (0.4 < EC_50_ µM < 8.8), especially on HEK293T.

**Table 2:** EC_50_ values of selected compounds **5**–**7** and TBB against four cell lines.

Compound	EC_50_ in µM ± SD			
	Normal cell lines ^1)^			
	NIH-3T3	WI-38	HEK293T	MEF
**5a**	> 10	> 10	> 10	> 10
**5b**	> 10	> 10	> 10	4.4 ± 0.3
**5c**	> 10	> 10	> 10	> 10
**5f**	> 10	> 10	> 10	> 10
**5g**	> 10	> 10	> 10	> 10
**6a**	> 10	> 10	> 10	> 10
**6b**	> 10	> 10	> 10	> 10
**6c**	> 10	> 10	> 10	> 10
**6d**	> 10	> 10	> 10	> 10
**6e**	> 10	> 10	> 10	> 10
**6f**	> 10	> 10	> 10	> 10
**7a**	not soluble	not soluble	not soluble	not soluble
**7b**	> 10	> 10	> 10	> 10
**7c**	> 10	> 10	1.6 ± 0.2	> 10
**7d**	> 10	> 10	4.9 ± 2.4	8.5 ± 0.4
**7f**	> 10	> 10	4.4 ± 2.2	> 10
**7g**	1.5 ± 0.8	2.5 ± 0.4	0.40 ± 0.02	> 10
ellagic acid	> 10	> 10	> 10	> 10
emodin	> 10	> 10	> 10	> 10
TBB	> 10	> 10	> 10	> 10

^1)^ Cells were cultured in presence of various concentrations of the respective conjugate. After 96 h, cytotoxicity was evaluated using the WST-1 assay; see Experimental Section 3.3.4.

**Figure 7:**
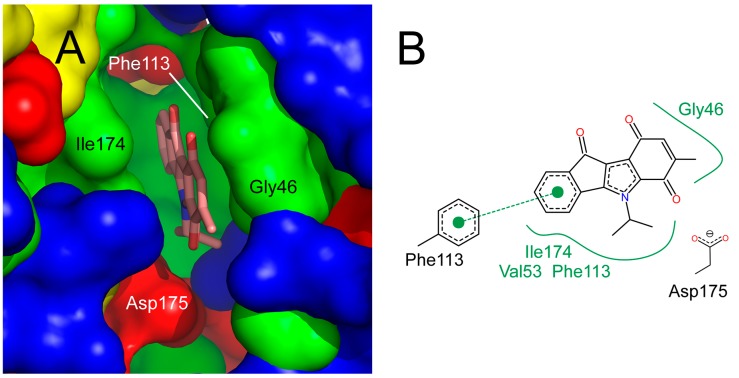
(**A**) Predicted binding mode of **7a** in the binding pocket of CK2. Green: hydrophobic surface patches, yellow: hydrophilic surface patches, blue (red): positively (negatively) polarized surface patches. Val53 and Ile174 shown in panel B are hidden behind Gly46. (**B**) Schematic view of the interactions between **7a** and neighboring amino acids (modified from a PoseView drawing [28]).

In order to understand observed differences in the inhibitory activity of compounds **5a**, **7a**, **5h** (R_1_ = R_2_ = H) [13] and **7h** (R_1_ = R_2_ = H) [14] (Table S1 in the supplementary data), the compounds were structurally aligned with the program vROCS [24] onto an inhibitor with pyridocarbazolone scaffold co-crystallized with CK2 (PDB code: 3OWJ [12]). Subsequently, the compounds were energy minimized in the presence of the enzyme with the program Moloc [26], keeping all protein atoms fixed. For this the co-crystallized water molecule 429 was removed, as it clashed with the ligands (distance ≤ 2.2 Å); in contrast, water molecule 382 was kept as its distance to the ligands was ~ 4 Å. In addition, the minimization was performed with all binding site residues within 5 Å of the ligands (L45-R47, S51, V53, V66, K68, I95, F113-V116, H160-N161, M163, and I174-W176) allowed to move, in order to investigate if the ligand binding would cause conformational changes within the binding site. The resulting protein structures showed only minor differences (Figure S1 in the supplementary data) that are within the experimental uncertainty of the structure determination (root mean-square deviation of the heavy atoms of the binding pocket < 0.4 Å); these differences are mainly caused by optimized intramolecular hydrogen-bonding of the CK2 residues. Thus, we focused on the binding modes of **5a**, **7a**, **5h** and **7h** obtained with fixed protein atoms for a better comparison to the crystal structure. The obtained binding modes of **5a**, **7a**, **5h** and **7h** agree very well with the one of the co-crystallized inhibitor (Figure S2 in the supplementary data). These binding modes provide explanations as to the observed differences in the inhibitory activity (Figure 7): (I) A carbonyl oxygen at position 6 points towards the negatively charged side chain of Asp175 resulting in the vicinity of two hydrogen bond acceptor groups. This unfavorable interaction increases the IC_50_ value of **7h** [14] by a factor of 15 compared to **5h** [13]. (II) Methyl substitution in position 7, first, decreases the polarity of the carbonyl group and, second, partially shields the group from solvation in the unbound state, making desolvation effects upon binding less costly. This results in an increase in inhibitory activity for 7-methyl derivatives of the paraquinone series as observed for **7h** compared to its methylated analogue **7a** (IC_50_ values 5.55 and 0.43 µM, respectively). (III) The methyl group also makes contacts with the C_α_ atom of Gly46 (Figure 7). A complete burial of a methyl group can increase a binding constant by up to 10-fold [27]_._ Here, the methyl group becomes only partially buried, which can explain the only two-fold increase of the inhibitory activity of **5a** compared to **5h**.

## 3. Experimental Section

### 3.1. General

Melting points were measured in capillary tubes using a BUCHI 510 apparatus and were uncorrected. IR spectra were recorded on a Perkin Elmer 1310 spectrometer and a Spectrum One spectrometer using KBr pellets (ν cm^−1^). ^1^H-NMR and ^13^C-NMR spectra (broadband decoupling and DEPT-135) were recorded on a Brucker Avance 400 (400 MHz for ^1^H and 100 MHz for ^13^C) or a Brucker Avance 500 spectrometer (500 MHz for ^1^H and 125 MHz for ^13^C) using CDCl_3_ or DMSO-d6 as solvents. NMR analysis of compounds **4**–**7** was performed with the same experiments described in [22]. Chemical shifts (δ) are referred to that of the solvent. Low resolution mass spectra were recorded on an Agilent 1290 Infinity system equipped with an Agilent 1260 DAD detector and an Agilent 6120 Quadrupole mass detector with an ESI source in positive mode. HRMS spectra were performed on a Q-Tof Micro Water with an ESI source in positive mode. Flash chromatography was performed on 230-400-mesh silica.

### 3.2. Synthesis

#### 3.2.1. General Procedure: Synthesis of Enaminones **2a**–**g**

Isopropylamine and the corresponding substituted cyclohexane-1,3-dione were dissolved in toluene in equimolar amounts. The reaction mixture was refluxed with a Dean-Stark trap until separation of H_2_O ceased (*ca.* 6 h). Solvent was removed in vacuo and the residual solid or viscous oil was treated with ethyl acetate to give after filtration and drying the product as a powder.

*5-Methyl-3-isopropylaminocyclohex-2-enone* (**2a**). Pale yellow powder; 75% yield; mp 106.7 °C; IR (KBr ν cm^−1^) 3250 (N-H), 1552 (C=O); ^1^H-NMR (DMSO-*d_6_*_,_ δ ppm, *J* Hz) 6.82 (d, 1H, *J =* 6.04, N-H), 4.78 (s, 1H, H-2), 3.46 (m, 1H, NCHMe_2_), 2.28 (m, 1H, H-6), 2.03–2.11 (m, 3H, H-4 and H-6), 1.82 (m, 1H, H-5), 1.10 (d, *J* = 4.3, 6H, NCHMe_2_), 0.95 (d, 3H, *J* = 5.79, CH_3_-5); ^13^C-NMR (DMSO-*d_6_*, δ ppm) 194.1 (C-1), 162.4 (C-3), 94.2 (C-2), 44.7 (C-6), 43.0 (C-5), 36.6 (C-4), 28.7 (NCHMe_2_), 21.6 (CH_3_), 21.5 (CH_3_), 20.8 (CH_3_); MS- ESI (*m/z*) 168.2 [M+H]^+^.

*5-Isopropyl-3-isopropylaminocyclohex-2-enone* (**2b**)*.* Pale yellow powder; 74% yield; mp 125.2 °C; IR (KBr, ν cm^−1^) 3259 (N-H) ,1538 (C=O); ^1^H-NMR (DMSO-*d_6_*_,_ δ ppm, *J* Hz) 6.86 (d, 1H, *J =* 6.5, N-H), 4.78 (s, 1H, H-2), 3.46 (m, 1H, NCHMe_2_), 2.30 (d, 1H, *J =* 13.8, H-6), 2.08 (m, 2H, H-4 and H-6), 1,87 (dd, 1H, *J =* 12.3 and 15.6, H-4), 1.65 (m, 1H, CHMe_2_-5 or H-5), 1.49 (m, 1H, H-5 or CHMe_2_-5), 1.11 (d, 3H, *J =* 6.2 NCHMe_2_), 1.09 (d, *J* = 6.3, 3H, NCHMe_2_), 0.87 (d, *J* = 6.5, 6H, CHMe_2_-5); ^13^C-NMR (DMSO-*d_6_*, δ ppm) 195.1 (C-1), 163.7 (C-3), 95.7 (C-2), 43.9 (NCHMe_2_), 40.9 (C-6), 40.9 (C-5), 33.2 (C-4), 32.1 (CHMe_2_), 22.5 (CH_3_), 22.3 (CH_3_), 20.3 (2 CH_3_); MS-ESI (*m/z*) 391.5 [2 M+H]+, 196.3 [M+H]^+^.

*3-Isopropylamino-5-phenylcyclohex-2-enone* (**2c**)*.* Pale yellow powder; quantitative yield; mp 145 °C; IR (KBr, ν cm^−1^) 3225 (N-H), 1537 (C=O); ^1^H-NMR (DMSO-*d_6_*_,_ δ ppm, *J* Hz) 7.28–7.40 (m, 5H, Ar-H), 7.02 (d, 1H, *J =* 6.8, N-H), 4.98 (s, 1H, H-2), 3.60 (m, 1H, NCHMe_2_), 3.28 (m, 1H, H-5), 2.69 (m, 1H, H-6), 2,52 (m, 2H, H- 4), 2.31 (dd, 1H, *J =* 3.5 and 15.9, H-6 ), 1.21 (d, 3H, *J =* 6.5, CH_3_ ), 1.19 (d, 3H, *J =* 6.3, CH_3_ ); ^13^C-NMR (DMSO-*d_6_*, δ ppm) 194.2 (C-1), 163.1 (C-3), 144.9 (C-1′), 129.2 (C-2′ and C-6′), 127.6 (C-3′ and C-5′), 126.1 (C-4′), 95.2 (C-2), 44.6 (C-6), 44.0 (NCHMe_2_), 40.2 (C-5), 36,7 (C-4), 22.5 (CH_3_), 22,3 (CH_3_); MS-ESI (*m/z*) 481.3 [2 M+Na]+, 230.1 [M+H]^+^.

*5-(4-Fluorophenyl)-3-isopropylaminocyclohex-2-enone* (**2d**)*.* Pale yellow powder; quantitative yield; mp 155 °C; IR (KBr, ν cm^−1^) 3674 (N-H), 1562 (C=O); ^1^H-NMR (DMSO-*d_6_*_,_ δ ppm, *J* Hz) 7.32 (m, 2H, Ar-H), 7.13–7.06 (m, 3H, Ar-H and N-H), 4.77 (s,1H, H-2 ), 3.30–3.15 (m, 2H, NCHMe_2_ and H-5), 2.32–2.39 (dd, 2H, *J =* 11.6 and 15.8, H- 4 and H-6 ), 2.17–2.22 (dd, 2H, *J =* 4.3 and 15.8, H-4 and H-6), 1.17 (d, 6H, *J =* 6.3, 2 CH_3_ ); ^13^C-NMR (DMSO-*d_6_*, δ ppm) 190.9 (C-1), 161.2 (d, ^1^*J*_C-F_ = 241, C-4′), 142.1 (d, ^4^*J*_C-F_ = 2.2, C-1′), 142.1 (C-3), 129.2 (d, ^3^*J*_C-F_ = 7.3, C-2′ and C-6′), 115.4 (d, ^2^*J*_C-F_ = 21.3, C-3′ and C-5′), 101.4 (C-2), 43.43 (C-4 and C-6), 43.1 (NCHMe_2_), 40.1 (C-5), 22.0 (2 CH_3_); MS-ESI (*m/z*) 270.1 [M+Na]^+^, 248.1 [M+H]^+^.

*5-Furan-2-yl-3-isopropylaminocyclohex-2-enone* (**2e**)*.* Pale yellow powder; 99% yield; mp 120 °C; IR (KBr, ν cm^−1^) 3258 (N-H), 1537 (C=O); ^1^H-NMR (DMSO-*d_6_*_,_ δ ppm, *J* Hz) 7.55 (d, 1H, *J =* 1.76, H-5′), 7.00 (d, 1H, *J =* 7.0, N-H), 6.37 (dd, 1H, *J =* 2.0 and 3.3, H-4′), 6.11 (d, 1H, *J =* 3.3, H-3′), 4.85 (s, 1H, H-2 ), 3.5 (m, 1H, H-5), 3.31 (m, 1H, NCHMe_2_), 2.65 (dd, 1H, *J =* 4.0 and 15.9, H-4 or H-6), 2.51 (m, 1H, H- 4 or H 6), 2.40 (dd, 1H, *J =* 4.5 and 16.1, H-4 or H-6), 2.30 (dd, 1H, *J =* 10.3 and 16.1, H-4 or H-6), 1.12 (d, 3H, *J =* 6.3, CH_3_ ), 1.10 (d, 3H, *J =* 6.3, CH_3_); ^13^C-NMR (DMSO-*d_6_*, δ ppm) 193.0 (C-1), 162.1 (C-3), 157.6 (C-2′) 142.1 (C-5′), 110.9 (C-3′ or C-4′), 104.9 (C-4′ or C-3′), 95.1 (C-2), 43.8 (CHMe_2_), 41.3 (C-4 or C-6), 33.8 (C-6 or C-4), 33.3 (C-5), 22.2 (CH_3_), 22.3 (CH_3_); MS-ESI (*m/z*) 461.3 [2 M+Na]+, 220.1 [M+H]^+^.

*3-Isopropylamino-6-methyl-cyclohex-2-enone* (**2f**). Pale yellow powder; 82% yield; mp 106.5 °C; IR (KBr ν cm^−1^) 3261 (N-H), 1540 (C=O); ^1^H-NMR (DMSO-*d_6_*_,_ δ ppm, *J* Hz) 6.76 (d, 1H, *J =* 6.4, N-H ), 4.76 (s, 1H, H-2), 3.46 (m, 1H, NCHMe_2_), 2.26–2.39 (m, 2H, H-4 and H-6), 2.07 (m, 1H, H-4), 1.2 (m, 1H, H-5), 1.49 (m, 1H, H-5), 1.10 (d, 3H, *J* = 6.4, NCHMe_2_), 1.09 (d, 3H, *J* = 6.4, NCHMe_2_), 0.98 (d, 3H, *J* = 6.95, CH_3_-6); ^13^C-NMR (DMSO-*d_6_*, δ ppm) 196.5 (C-1), 162.5 (C-3), 94.0 (C-2), 43.0 (NCHMe_2_), 38.8 (C-6), 29.6 (C-5), 21.65 (NCHMe_2_), 21.7 (NCHMe_2_), 16.0 (CH_3_-6); MS-ESI (*m/z*) 168.2 [M+H]^+^.

*3-Isopropylamino-6-isopropylcyclohex-2-enone* (**2g**)*.* Pale yellow powder; 78% yield; mp 121 °C; IR (KBr, ν cm^−1^) 3268 (N-H), 1572 (C=O); RMN ^1^H (DMSO-*d_6_*_,_ δ ppm, *J* Hz) 6.74 (d, 1H, *J =* 6.7, N-H), 4.78 (s, 1H, H-2), 3.44 (m, 1H, NCHMe_2_), 2.4–2.2 (m, 3H, CHMe_2_ and H-6 and H-4), 1.75–1.86 (m, 2H, H-5 and H-4), 1.57 (m, 1H, H-5), 1.10 (d, 3H, *J =* 4.7, NCH(Me)_2_), 1.07 (d, 3H, *J =* 4.7, NCHMe_2_), 0.87 (d, 3H, *J =* 7.1, CHMe_2_-6), 0.70 (d, 3H, *J =* 6.8, CHMe_2_-6); ^13^C-NMR (DMSO-*d_6_*, δ ppm) 195.33 (C-1), 162.25 (C-3), 95.06 (C-2), 49.92 (NCHMe_2_), 42.96 (C-6), 27.98 (C-4), 25.70 (CHMe_2_-6), 21.65 (CH_3_), 21.53 (CH_3_), 21.31 (C-5), 20.54 (CH_3_), 18.03 (CH_3_); MS-ESI (*m/z*) 196.2 [M+H]^+^.

#### 3.2.2. General Procedure: Synthesis of Substituted 4*b*,9*b*-Dihydroxy-4*b*,5,6,7,8,9*b*-hexahydroindeno[1,2-*b*]indole-9,10-diones **4a**–**g**

Equimolar amounts of the corresponding enaminone **2a**–**g** and ninhydrin **3** were dissolved in methanol and stirred at room temperature for 22 h. Solvent was removed in vacuo and the residual solid or viscous oil was treated with diethyl ether to give the product as a powder after filtration and drying. A further amount was recovered from the mother liquor.

*4b,9b-Dihydroxy-5-isopropyl-7-methyl-4b,5,6,7,8,9b-hexahydroindeno[1,2-b]indole-9,10-dione* (**4a**). Yellow powder; 95% yield; mp 198.9 °C; IR (KBr ν cm^−1^) 3320 (OH), 3189 (OH), 1742 (C=O); ^1^H-NMR (CHCl_3,_ δ ppm, *J* Hz) 7.83 and 7.80 (m, 2H, H-1 and H-4), 7.71 and 7.69 (m, 1H, H-3), 7.51 and 7.49 (m, 1H, H-2), 5.28 (br s, 2H, 2 OH), 4.63 (m, 1H, NCHMe_2_), 2.78 (dd, 1H, *J* = 4.1 and 16.5, H-6), 2.57 (dd, 1H, *J* = 3.3 and 17, H-6), 2.33 (m, 1H, H-7), 2.29 (m, 1H, H-6), 2.27 (m, 1H, H-8), 2.04 (m, 3H, H-6 and H-7 and H-8), 1.93 (m, 1H, H-8), 1.57 (d, 1H, *J* = 7, NCHMe_2_), 1.53 (d, 1H, *J* = 7.1, NCHMe_2_), 1.35 (d, 3H, *J* = 7.3, NCHMe_2_), 1.32 (d, 3H, *J* = 7.2, NCHMe_2_), 1.03 (d, *J* = 6.2, 3H, CH_3_), 1.00 (d, *J* = 6.5, 3H, CH_3_); ^13^C-NMR (CHCl_3_, δ ppm) 197.7 and 197.1 (C-10), 191.7 and 191.3 (C-9), 165.9 and 165.7 (C-5a), 148.5 and 148.0 (C-4a), 135.7 (C-3), 135.4 and 135.3 (C-10a), 130.4 and 130.4 (C-2), 124.9 and 124.7 (C-1 or C-4), 124.1 and 123.7 (C-1 or C-4), 105.6 and 105.4 (C-9a), 96.3 and 96.0 (C-4b), 82.7 and 82.3 (C-9b), 46.4 and 46.1 (CHMe_2_), 44.6 and 45.1 (C-8), 33.2 and 33.1 (C-6), 30.2 and 29.8 (C-7), 23.6 and 23.3 (CH_3_), 22.8 and 22.7 (CH_3_), 21.4 and 21.1 (CH_3_); MS-ESI (*m/z*) 677.3 [2 M+Na]+, 350.1 [M+Na]^+^, 328.2 [M+H]^+^.

*4b,9b-Dihydroxy-5,7-diisopropyl-4b,5,6,7,8,9b-hexahydroindeno[1,2,b]indole-9,10-dione* (**4b**). Yellow powder; quantitative yield; mp 200.8 °C; IR (KBr, ν cm^−1^) 3252 (OH), 3180 (OH), 1728 (C=O); for complete NMR data see [22]; MS-ESI (*m/z*) 733.7 [2 M+Na]+, 378.4 [M+Na]^+^, 356.3 [M+H]^+^.

*4b,9b-Dihydroxy-5-isopropyl-7-phenyl-4b,5,6,7,8,9b-hexahydroindeno[1,2-*b*]indole-9,10-dione* (**4c**). Yellow powder; quantitative yield; mp 181.1 °C; IR (KBr, ν cm^−1^) 1719 (C=O); ^1^H-NMR (DMSO-*d_6_*_,_ δ ppm, *J* Hz) 7.99 (m, 1H, Ar-H), 7.83 (m, 1H, Ar-H), 7.75 (m, 1H, Ar-H), 7.63 (m, 1H, Ar-H), 7.39–7.2 (m, 5H, Ar-H), 6.88 and 6.78 (2 s, 1H, OH), 5.81 and 5.72 (2 s, 1H, OH), 4.64 (m, 1H, NCHMe_2_), 3.22 and 3.13 (2 m, 1H, H-7), 2.92 (m, 1H, H-6 or H-8), 2.80 (m, 1H, H-8 or H-6), 2.47 (m, 1H, H-6 or H-8), 2.21 (m, 1H, H-8 or H-6), 1.50 and 1.43 (2 d, *J* = 6.8, 3H, CH_3_), 1.28 and 1.26 (2 d, 3H, *J =* 7.3 and 8.3, CH_3_); ^13^C-NMR (DMSO-*d_6_*, δ ppm) 198.7 and 198.1 (C-10), 188.7 and 188.2 (C-9), 164.7 (C-5a), 149.4 and 148.7 (C-4a), 144.6 and 144.5 (C-1′), 136.5 and 136.4 (C-3), 135.9 and 135.7 (C-10a), 131.2 and 131.1 (C-2), 129.3 (C-3′ and C-5′), 128.0 and 128.0 (C-2′and C-6′), 127.5 and 127.5 (C-4′), 125.7 and 125.3 (C-4), 124.3 and 124.0 (C-1), 105.8 (C-9a), 97.1 and 96.9 (C-4b), 84.0 and 83.7 (C-9b), 46.0 and 45.8 (NCHMe_2_), 45.5 and 45.1 (C-8), 41.3 and 40.5 (C-7), 32.3 and 31.6 (C-6), 23.8 and 23.3 (CH_3_), 23.5 and 23.4 (CH_3_); MS-ESI (*m/z*) 801.4 [2 M+Na]+, 412.1 [M+Na]^+^, 390.2 [M+H]^+^.

*4b,9b-Dihydroxy-7-(4-Fluoro-phenyl)-5-isopropyl-4b,5,6,7,8,9b-hexahydroindeno[1,2-*b*]indole-9,10-dione* (**4d**). Yellow powder; 75% yield; mp 161.1 °C; IR (KBr, ν cm^−1^) 3150 (OH), 3186 (OH), 1741 (C=O); for complete NMR data see [22]; MS-ESI (*m/z*) 408.2 [M+H]^+^; HRMS-ESI (*m/z*) [M+H]^+^ calcd for C_24_H_23_FNO_4_ 408.1606, found 408.1587.

*Isopropylammonium* *4-(4-fluorophenyl)-1-(2-hydroxy-1,3-dioxo-2,3-dihydro-1H-inden-2-yl)-2,6-dioxocyclohexan-1-ide* (**4d’**). Pale yellow powder; 10% yield; mp 161.1 °C; IR (KBr ν cm^−1^) 3150 (OH), 1741 (C=O), 1706 (C=O); ^1^H-NMR (DMSO-*d_6_*_,_ δ ppm, *J* Hz) 7.87 (m, 2H, 2 H-Phe), 7.82 (m, 2H, 2 H-Phe), 7.66 (br s, 2H, OH), 7.35 (m, 2H, 2 H-Phe), 7.10 (m, 2H, 2 H-Ar), 3.23 (m, 1H, NCHMe_2_), 3.22 (m; 1H, CH-Ar), 2.38 (dd, 2H, *J* = 11.9 and 16.1, CH_2_), 2.13 (dd, 2H, *J* = 4.3 and 16.1, CH_2_), 1.15 (d, 6H, *J* = 6.4, 2 CH_3_); ^13^C-NMR (DMSO-*d_6_*, δ ppm) 202.4 (2 C=O), 190.1 (2 C=O), 160.6 (d, ^4^*J*_C-F_ = 240.2, C-4′), 141.0 (d, *J* = 2.2, C-1′), 140.0 (C-OH), 134.8 (C-2 and C-3), 128.6 (d, ^3^*J*_C-F_ = 8.0, C-2′ and C-6′), 122.4 (C-1 and C-4), 114.8 (d, ^2^*J*_C-F_ = 20.8, C-3′ and C-5′), 105.1 (Cq), 80.0 (C-OH), 42.9 (2 CH_2_ and CHPhe), 38.7 (NCHMe_2_), 20.2 (NCHMe_2_); MS-ESI (*m/z*) 755.2 [2 M+Na]+, 367.1 [M+H]^+^; HRMS-ESI (*m/z*) [M+Na]^+^ calcd for C_21_H_15_NFNaO_5_ 389.0792, found 389.0796.

*7-Furan-2-yl-4b,9b-dihydroxy-5-isopropyl-4b,5,6,7,8,9b-hexahydroindeno[1,2-*b*]indole-9,10-dione* (**4e**). Yellow powder; quantitative yield; mp 135 °C; IR (KBr, ν cm^−1^) 3159 (OH), 3150 (OH), 1719 (C=O); ^1^H-NMR (DMSO-*d_6_*_,_ δ ppm, *J* Hz) 7.99 (m, 1H, H-1), 7.82 (m, 1H, H-4), 7.74 (m, 1H, H-3), 7.65–7.5 (m, 2H, Ar-H and H-5′), 6.90 and 6.81 (2 s, 1H, OH), 6.40 and 6.32 (2 dd, 1H, *J =* 1.8, and 3, H-4′), 6.23 and 6.08 (2 d, 1H, *J =* 3.2, H-3′), 5.79 and 5.70 (2 s, 1H OH), 4.65 (m, 1H, NCHMe_2_), 3.43 and 3.33 (2 m, 1H, H-7), 3.09 (dd, 1H, *J =* 4.3 and 16.9, H-6), 2.68 (m, 1H, H-8), 2.47–2.27 (m, 2H, H-6 and H-8), 1.52 and 1.47 (2 d, 3H, *J =* 7, CH_3_), 1.32 and 1.30 (2 d, 3H, *J =* 4 and 4.3, CH_3_); ^13^C-NMR (DMSO-*d_6_*, δ ppm) 198.1 and 197.7 (C-10), 187.3 and 186.9 (C-9), 163.5 and 163.2 (C-5a), 156.9 (C-2′), 148.8 and 148.3 (C-4a), 142.1 and 142.1 (C-5′), 136.1 and 136.0 (C-3), 135.5 and 135.3 (C-10a), 130.8 and 130.7 (C-2), 125.0 and 125.2 (C-1), 123.9 and 123.6 (C-4), 110.9 and 110.8 (C-3′ or C-4′), 105.5 and 105.2 (C-4′ or C-3′), 96.5 and 96.4 (C-4b), 83.5 and 83.3 (C-9b), 45.6 and 45.4 (NCHMe_2_), 42.1 and 41.5 (C-8), 34.0 and 33.4 (C-7), 29.4 and 29.2 (C-6), 23.3 and 23.1 (CH_3_), 23.0 and 23.0 (CH_3_); MS-ESI (*m/z*) 781.3 [2 M+Na]^+^, 402.1 [M+Na]^+^, 380.2 [M+H]^+^.

*4b,9b-Dihydroxy-5-isopropyl-8-methyl-4b,5,6,7,8,9b-hexahydro-indeno[1,2-*b*]indole-9,10-dione* (**4f**). Yellow pale powder; 46% yield; mp 198.9 °C; IR (KBr ν cm^−1^) 3190 (OH), 3160 (OH), 1725 (C=O); ^1^H-NMR (DMSO-*d_6_*_,_ δ ppm, *J* Hz) 7.94 and 7.92 ( 2 d, 1H, *J* = 7.8, H-1), 7.78 (m, 1H, H-4), 7.70 and 7.67 (2 d, 1H, *J* = 7.5, H-3), 7.57 (m, 1H, H-2), 6.71 (s, 1H, OH minor - 45%), 6.64 (s, 1H, OH major - 55%), 5.60 (s, 1H, OH major), 5.59 (s, 1H, OH minor), 4.56 (m, 1H, CHMe_2_), 2.76 (m, 1H, H-8 major), 2.66 (m, 1H, H-6 or H-7 minor), 2.58 (m, 1H, H-8 minor), 2.44 (m, 1H, H-6 or H-7 major), 2.1 (m, 1H, H-6 or H-7 minor), 2.04 (m, 1H, H-6 or H-7 major), 1.92 (m, 1H, H-6 or H-7 major), 1.57 (m, 1H, H-7 major), 1.45 (d, 3H, *J* = 7.0, CH_3_ major), 1.42 (d, 3H, *J* = 7.0, CH_3_ minor), 1.41 (m, 1H, H-7 minor), 1.24 (d, 3H, *J* = 7.0, CH_3_ major), 1.21 (d, 3H, *J* = 7.2, CH_3_ minor), 0.97 (d, 3H, *J* = 6.8, CH_3_ major), 0.93 (d, 3H, *J* = 6.9, CH_3_ minor); ^13^C-NMR (DMSO-*d_6_*, δ ppm) 197.1 and 196.6 (C-10), 190.7 and 190.4 (C-9), 163.4 and 163.3 (C-5a), 147.7 and 147.3 (C-4a), 134.7 and 134.7 (C-3), 134.2 and 134.0 (C-10a), 129.5 and 129.4 (C-2), 124.0 and 123.6 (C-4), 122.6 and 122.3 (C-1), 103.8 and 103.5 (C-9a), 95.1 and 95.0 (C-4b), 82.5 and 82.4 (C-9b), 44.2 and 44.0 (CHMe_2_), 39.6 and 39.1 (C-8), 30.2 and 29.7 (C-6), 23.7 and 23.4 (C-7), 22.7 and 22.5 (CH_3_), 22.3 and 22.2 (CH_3_), 15.7 and 15.4 (CH_3_-8); MS-ESI^+^ (*m/z*) 677.3 [2 M+Na]+, 350.2 [M+Na]^+^, 328.2 [M+H]^+^.

*4b,9b-Dihydroxy-5,8-diisopropyl-4b,5,6,7,8,9b-hexahydroindeno[1,2-*b*]indole-9,10-dione* (**4g**). Yellow powder; 55% yield; mp 220 °C; IR (KBr, ν cm^−1^) 3675 (OH), 3158 (OH), 1719 (C=O); ^1^H-NMR (DMSO-*d_6_*_,_ δ ppm, *J* Hz) 7.93 (m, 1H, H-1), 7.80 (m, 1H, H-4), 7.70 (m, 1H, H-3), 7.58 (m, 1H, H-2), 6.75 (2 s, 1H, OH), 5.64 (br s, 1H, OH), 4.55 (m, 1H, NCHMe_2_), 2.84 (m, 1H, H-8), 2.80 (m, 1H, H-8), 2.60 (m, 2H, H-6 and H-8), 2.37 (m, 1H, CHMe_2_-8), 2.17 (m, 1H, CHMe_2_-8), 1.82 (m, 2H, H-7 and H-6), 1.64 (m, 1H, H-7), 1.51 (m, 1H, H-7), 1.45 (d, 3H, *J =* 7.0, CH_3_), 1.41 (d, 3H, *J =* 7.0, CH_3_), 1.23 (d, 3H, *J =* 7.0, CH_3_), 1.20 (d, 3H, *J =* 7.0, CH_3_), 0.86 (d, 3H, *J =*7.0, CH_3_), 0.80 (d, 3H, *J =* 7.0, CH_3_), 0.74 (d, 3H, *J =* 6.8, CH_3_), 0.64 (d, 3H, *J =* 6.8, CH_3_); ^13^C-NMR (DMSO-*d_6_*, δ ppm) 197.9 and 197.3 (C-10), 191.0 and 189.7 (C-9), 163.8 and 163.6 (C-5a), 148.4 and 147.9 (C-4a), 135.4 and 135.3 (C-3), 134.9 and 134.6 (C-10a), 130.1 and 130.0 (C-2), 124.6 and 124.3 (C-1), 123.2 and 123.0 (C-4), 105.3 and 105.0 (C-9a), 95.8 and 95.6 (C-4b), 83.3 and 82.9 (C-9b), 50.5 and 49.9 (NCHMe_2_), 44.8 and 44.6 (C-8), 25.9 and 25.2 (CHMe_2_-8), 23.8 and 23.1 (C-7), 22.7 and 22.4 (CH_3_), 22.2 and 22.1 (CH_3_), 21.9 and 21.8 (C-6), 20.6 and 20.5 (CH_3_), 18.4 and 18.0 (CH_3_); MS-ESI (*m/z*) 356.2 [M+H]^+^.

#### 3.2.3. Synthesis of *N*,*N*,*N′*,*N′*-Tetraisopropylthionylamide (TIPTA) and *N*,*N*,*N′*,*N′*-Tetraethylthionylamide (TETA)

Diisopropylamine (161.01 mmol) was introduced into 150 mL of distilled diethyl ether at −40 °C. A thionyl chloride solution (4.25 mL) in diethyl ether (10 mL) was introduced dropwise during one hour with release of a white smoke. Temperature increased to −20 °C at the end of the addition. The viscous mixture was stirred for 3 h with the temperature reaching 0 °C. The mixture was then filtered under vacuum to remove the diisopropylammonium chloride precipitate and washed with diethyl ether. The filtrate was concentrated and evaporated to dryness to afford a white precipitate of TIPTA (85% yield; mp 57.1–58.2 °C).

*N*,*N*,*N′*,*N′*-tetraethylthionylamide (TETA) was prepared by the same method unless diisopropylamine was replaced by diethylamine (93% yield).

#### 3.2.4. General Procedure: Synthesis of Substituted 5,6,7,8-Tetrahydroindeno[1,2-*b*]indole-9,10-diones **5a**–**g**

The corresponding dihydroxy derivative **4a**–**g** was dissolved in 25 mL DMF and 5 mL acetic acid. After addition of TIPTA or TETA (1.5 eq.) the mixture was stirred for 23 h at room temperature and then the mixture was poured into ice water (200 mL). After 1.50 h the precipitate **5a**–**g** was recovered by filtration.

*5-Isopropyl-7-methyl-5,6,7,8-tetrahydroindeno[1,2-*b*]indole-9,10-dione* (**5a**). Orange powder; quantitative yield; mp 205.9 °C; IR (KBr, ν cm^−1^) 1710 (C=O), 1667 (C=O); ^1^H-NMR (CHCl_3,_ δ ppm, *J* Hz) 7.74 (d, 1H, *J* = 7.1, H-1), 7.23 (m, 1H, H-3), 7,11 (m, 2H, H-2 and H-4), 4.62 (sept, 1H, *J* = 7.1, NCH(Me)_2_), 2.92 (dd, 1H, *J* = 4.3 and 16.2, H-6), 2.54 (dd, 1H, *J* = 3.6 and 16.2, H-8), 2.50 (m, 1H, H-6), 2.39 (m, 1H, H-7), 2.20 (dd, 1H, *J* = 11.8 and 16.2, H-8), 1.65 (d, 3H, *J* = 7.3, NCH(Me)_2_), 1.64 (d, 3H, *J* = 7.0, NCH(Me)_2_), 1.17 (d, 3H, *J =* 6.5, CH_3_); ^13^C-NMR (CHCl_3_, δ ppm) 191.9 (C-9), 184.2 (C-10), 151.8 (C-4b), 148.7 (C-5a), 138.9 (C-10a), 135.4 (C-4a), 132.2 (C-3), 128.1 (C-2), 123,8 (C-1), 118.8 (C-4), 120.8 (C-9b), 117.4 (C-9a), 49.4 (N-CH(Me)_2_), 46.1 (C-8), 31.7 (C-6), 31.1 (C-7), 22.0 (CH_3_), 21.9 (CH_3_), 21.3 (CH_3_-7); MS-ESI (*m/z*) 609.3 [2 M+Na]+, 316.1 [M+Na]^+^, 294.1 [M+H]^+^; HRMS-ESI (*m/z*) [M+Na]^+^ calcd for C_19_H_19_NO_2_Na 316.1308, found 316.1312.

*5,7-Diisopropyl-5,6,7,8-tetrahydroindeno[1,2-*b*]indole-9,10-dione* (**5b**)*.* Orange powder; quantitative yield; mp 191 °C; IR (KBr, ν cm^−1^) 1710 (C=O), 1667 (C=O); for complete NMR data see [22]; MS-ESI (*m/z*) 665.8 [2 M+Na]+, 344.4 [M+Na]^+^, 322.4 [M+H]^+^; HRMS-ESI (*m/z*) [M+Na]^+^ calcd for C_21_H_23_NO_2_Na 344.1621, found 344.1616.

*5-Isopropyl-7-phenyl-5,6,7,8-tetrahydroindeno[1,2-*b*]indole-9,10-dione* (**5c**). Orange powder; 76% yield; mp 278 °C; IR (KBr, ν cm^−1^) 1703 (C=O), 1651 (C=O); ^1^H-NMR (CDCl_3_, δ ppm, *J* Hz) 7.40 (d, 1H, *J =* 6.8, H-1), 7.31 (m, 2H, Ar-H), 7.12 (m, 4H, Ar-H), 7.07 (m, 2H, Ar-H), 4.56 (sept, *J* = 6.7, 1H, NCHMe_2_), 3.46 (m, 1H, H-7), 3.08 (dd, 1H, *J* = 4.8 and 16.4, H-6), 2.93 (dd, 1H, *J* = 11.1 and 16.4, H-8), 2.69 (m, 2H, H-8 and H-6), 1.59 (d, 3H, *J =* 6.8, CH_3_), 1.58 (d, 3H, *J =* 7.0, CH_3_); ^13^C-NMR (CDCl_3_, δ ppm) 191.1 (C-9), 184.4 (C-10), 152.2 (C-4b), 148.3 (C-5a), 142.9 (C-1′), 139.0 (C-4a), 135.5 (C-10a), 132.4 (C-3), 129.0 (C-3′ and C-5′), 128.4 (C-2), 127.4 (C-4′), 127.0 (C-2′ and C-6′), 124.0 (C-1), 121.0 (C-9b), 119.0 (C-4), 117.6 (C-9a), 49.3 (N-CHMe_2_), 44.9 (C-8), 41.9 (C-7), 31.8 (C-6), 22.2 (CH_3_), 22.0 (CH_3_); MS-ESI (*m/z*) 733.3 [2 M+Na]+, 378.1 [M+Na]^+^, 356.2 [M+H]^+^; HRMS-ESI (*m/z*) [M+Na]^+^ calcd for C_24_H_21_NNaO_2_ 378.1465, found 378.1461.

*7-(4-Fluorophenyl)-5-isopropyl-5,6,7,8-tetrahydroindeno[1,2-*b*]indole-9,10-dione* (**5d**). Orange powder; 78% yield; mp 264 °C; IR (KBr, ν cm^−1^) 1701 (C=O), 1670 (C=O); for complete NMR data see [22]; HRMS-ESI (*m/z*) [M+Na]^+^ calcd for C_24_H_20_FNNaO_2_ 396.1370, found 396.1362.

*7-Furan-2-yl-5-isopropyl-5,6,7,8-tetrahydroindeno[1,2-*b*]indole-9,10-dione* (**5e**). Orange powder; 78% yield; mp 233.1 °C; IR (KBr, ν cm^−1^) 1705 (C=O), 1661 (C=O); ^1^H-NMR (CDCl_3_, δ ppm, *J* Hz) 7.38 (d, 1H, *J* = 7.0, H-1), 7.17 (m, 2H, 2 H-Ar), 7.05 (2H, H-Ar and H-5′), 6.31 (m, 1H, H-3′), 6.02 (d, *J* = 2.9, 1H, H-4′), 4.55 (m, 1H, NCHMe_2_), 3.54 (m, 1H, H-7), 3.17 (dd, 1H, *J* = 4.6 and 16.4, H-6 or H-8), 2.96 (dd, 1H, *J* = 9.7 and 16.3, H-8 or H-6), 2.77 (dd, 1H, *J* = 5.4 and 17.9, H-6 or H-8), 2.36 (dd, 1H, *J* = 10.9 and 16.4, H-6 or H-8), 1.57 (d, 3H, *J* = 6.8, CH_3_), 1.56 (d, 3H, *J* = 6.8, CH_3_); ^13^C-NMR (CDCl_3_, δ ppm) 190.2 (C-9), 184,2 (C-10), 155.7 (C-2′), 152.1 (C-4b), 147.2 (C-5a), 141.6 (C-5′), 138.9 (C-10a), 135.3 (C-4a), 132.2 (C-3), 128.3 (C-2), 124.0 (C-1), 118.8 (C-4), , 117.5 (C-9), 110.3 (C-3′ or C-4′), 105.2 (C-4′ or C-3′), 41.9 (C-8), 49.6 (NCHMe_2_), 34.9 (C-7), 28.6 (C-6), 22.05 (CH_3_), 21.9 (CH_3_); MS-ESI (*m/z*) 713.3 [2 M+Na]+, 368.1 [M+Na]^+^, 346.1 [M+H]^+^; HRMS-ESI (*m/z*) [M+Na]^+^ calcd for C_22_H_19_NNaO_3_ 368.1257, found 368.1258.

*5-Isopropyl-8-methyl-5,6,7,8-tetrahydroindeno[1,2-*b*]indole-9,10-dione* (**5f**). Orange powder; quantitative yield; mp 200 °C; ^1^H-NMR (DMSO-*d_6_*_,_ δ ppm, *J* Hz) 7.37–7.33 (m, 2H, Ar-H), 7.30 (d, 1H, *J =* 7.0, Ar-H), 7.17 (m, 1H, Ar-H), 4.72 (m, 1H, NCHMe_2_), 2.99 (m, 1H, H-6 or H-7), 2.91 (m, 1H, H-6 or H-7), 2.41 (m, 1H, H-8), 2.14 (m, 1H, H-6 or H-7), 1.88 (m, 1H, H-6 or H-7), 1.55 (d, 3H, *J* = 6.8, NCHMe_2_), 1.54 (d, 3H, *J* = 6.8, NCHMe_2_), 1.07 (d, 3H, *J* = 6.9, CH_3_); ^13^C-NMR (DMSO-*d_6_*, δ ppm) 193.6 (C-9), 183.3 (C-10), 151.2 (C-4b), 150.02 (C-5a), 138.0 (C-4a), 134.65 (C-10a), 132.8 (C-3), 128.2 (C-2), 122.9 (C-1), 120.4 (C-9b), 119.4 (C-4), 116.0 (C-9a), 46.1 (N-CH(Me)_2_), 40.6 (C-8), 30.3 (C-6), 22.2 (C-7), 21.4 (CH_3_), 21.2 (CH_3_), 14.8 (CH_3_-8); MS-ESI (*m/z*) 609.3 [2 M+Na]+ 316.2 [M+Na]^+^, 294.2 [M+H]^+^; HRMS-ESI (*m/z*) [M+H]^+^ calcd for C_19_H_19_ NO_2_Na 316.1308, found 316.1315.

*5,8-Diisopropyl-5,6,7,8-tetrahydroindeno[1,2-*b*]indole-9,10-dione* (**5g**)*.* Orange powder; 75% yield; mp 215 °C; IR (KBr, ν cm^−1^) 1738 (C=O), 1712 (C=O); ^1^H-NMR (CDCl_3,_ δ ppm, *J* Hz) 7.45 (m, 1H, Ar-H), 7.23 (m, 1H, Ar-H), 7.12–7.06 (m, 2H, Ar-H), 4.59 (m, 1H, NCHMe_2_), 2.94 (m, 1H, H-6 or H-7), 2.78 (m, 1H, H-6 or H-7), 2.47 (m, 1H, H-8), 2.17 (m, 2H, H-6 and H-7), 2.01 (m, 1H, H-6 or H-7), 1.63 (d, 3H, *J* = 7.0, NCHMe_2_), 1.62 (d, 3H, *J* = 7.0, NCHMe_2_), 0.98 (d, 3H, *J* = 6.9, CHMe_2_-8), 0.90 (d, 3H, *J* = 6.8, CHMe_2_-8); ^13^C-NMR (CDCl_3,_, δ ppm) 193.6 (C-9), 184.2 (C-10), 151.6 (C-4b), 148.1 (C-5a), 139.0 (C-4a), 135.4 (C-10a), 132.0 (C-3), 128.0 (C-2), 123.7 (C-1), 121.0 (C-9b), 118.6 (C-4), 118.6 (C-9a), 52.3 (N-CHMe_2_), 49.2 (C-8), 25.81 (CHMe_2_-8), 23.7 (C-6 or C-7), 22.9 (C-7 or C-6), 22.0 (CH_3_), 21.9 (CH_3_), 21.6 (CH_3_), 20.8 (CH_3_); MS-ESI (*m/z*) 665.4 [2 M+Na]+, 322.2 [M+H]^+^; HRMS-ESI (*m/z*) [M+H]^+^ calcd for C_21_H_24_NO_2_ 322.1802, found 322.1810.

#### 3.2.5. General Procedure: Synthesis of Substituted 9-Hydroxy-5*H*-indeno[1,2-*b*]indol-10-ones **6a**–**g**

The corresponding mono-keto derivative **5a**–**g** (4 mmol) was introduced into 15 mL diphenyl ether then Pd/C (150% m/m) was added. The reaction mixture was refluxed for 3 h. After cooling, methanol (50 mL) was added. The mixture was then filtrated on Celite and the solution concentrated. The obtained dark red oil was purified by column chromatography on silica gel (cyclohexane then ethyl acetate/cyclohexane: 1/2).

*9-Hydroxy-5-isopropyl-7-methyl-5H-indeno[1,2-*b*]indol-10-one* (**6a**). Dark red powder; 70% yield; IR (KBr, ν cm^−1^) 3181 (OH), 1701 (C=O); ^1^H-NMR (CDCl_3_, δ ppm, *J* Hz**)** 7.35 (d, 1H, *J =* 6.9, H-1), 7.22 (t, J = 7.5, 1H, H-3), 7.16 (d, *J* = 7.2, 1H, H-4), 7.11 (t, *J* = 7.3, 1H, H-2), 6.72 (s, 1H, H-6), 6.58 (s, 1H, OH), 6.49 (s, 1H, H-8), 4.81 (sept, *J* = 6.9, 1H, NCHMe_2_), 2.39 (s, 3H, CH_3_-7), 1.70 (d, 6H, *J*= 7.0, NCHMe_2_); ^13^C-NMR (CDCl_3_, δ ppm) 186.1 (C-10), 155.3 (C-4b), 149.7 (C-9), 142.9 (C-5a), 140.6 (C-10a), 136.5 (C-4a), 135.7 (C-7), 132.2 (C-3), 129.1 (C-2), 123.3 (C-1), 119.8 (C-4), 111.4 (C-9a), 109.2 (C-8), 104.9 (C-6), 49.5 (NCHMe_2_), 22.2 (CH_3_), 21.8 (2 CH_3_); MS-ESI (*m/z*) 605.3 [2 M+Na]+, 314.1 [M+Na]^+^, 292.1 [M+H]^+^; HRMS-ESI (*m/z*) calcd for C_19_H_17_NNaO_2_ 314.1151, found 314.1153.

*9-Hydroxy-5,7-diisopropyl-5H-indeno[1,2-*b*]indol-10-one* (**6b**)*.* Dark red powder; 89% yield; mp 188.1 °C; IR (KBr, ν cm^−1^) 3411 (OH), 1653 (C=O); for complete NMR data see [22]; MS-ESI (*m/z*) 661.7 [2 M+Na]+, 342.4 [M+Na]^+^, 320.4 [M+H]^+^; HRMS-ESI (*m/z*) [M+H]^+^ calcd for C_21_H_22_NO_2_ 320.1645, found 320.1649.

*9-Hydroxy-5-isopropyl-7-phenyl-5H-indeno[1,2-*b*]indol-10-one* (**6c**). Dark red powder; 56% yield; mp 198 °C; IR (KBr, ν cm^−1^) 3396 (OH), 2924 (CH), 1659 (C=O); ^1^H-NMR (CDCl_3_, δ ppm, *J* Hz) 7.61 (m, 2H, Ar-H), 7.47–7.41 (m, 3H, Ar-H), 7.35 (m, 1H, Ar-H), 7.29–7.22 (m, 2H, Ar-H), 7.17 (m, 1H, Ar-H), 7.19 (s, 1H, H-6), 6.93 (s, 1H, H-8), 6.73 (s, 1H, OH), 4.90 (sept, *J* = 7, 1H, NCHMe_2_), 1.76 (d, 6H, *J* = 7.0, 2 CH_3_); ^13^C-NMR (CDCl_3_, δ ppm) 186.0 (C-10), 156.0 (C-4b), 150.1 (C-9), 143.10 (C-5a), 141.8 (C-10a), 140.7 (C-4a), 139.3 (C-1′), 136.4 (C-7), 132.4 (C-3), 129.4 (C-2), 128.8 (C-3′ and C-5′), 127.3 (C-2′ and C-6′), 127.2 (C-4′), 123.6 (C-1), 120.1 (C-4), 115.7 (9b), 112.8 (C-9a), 107.6 (C-8), 103.7 (C-6), 49.8 (NCHMe_2_), 21.9 (2 CH_3_); MS-ESI (*m/z*) 730.3 [2 M+Na]+, 376.2 [M+Na]^+^, 354.1 [M+H]^+^; HRMS-ESI (*m/z*) [M+Na]^+^ calcd for C_24_H_19_NNaO_2_ 376.1308, found 376.1300.

*7-(4-Fluorophenyl)-9-hydroxy-5-isopropyl-5H-indeno*[1,2-*b*]*indol-10-one* (**6d**)*.* Dark red powder; 52% yield; mp 208.1 °C; IR (KBr, ν cm^−1^) 3391 (OH), 1659 (C=O); for complete NMR data see [22]; 394 [M+Na]^+^, 372 [M+H]^+^; HRMS-ESI (*m/z*) [M+H]^+^ calcd for C_24_H_19_FNO_2_ 372.1394, found 372.1377.

*7-Furan-2-yl-9-hydroxy-5-isopropyl-5H-indeno*[1,2-*b*]*indol-10-one* (**6e**)*.* Dark red powder; 50% yield; mp 191 °C; IR (KBr, ν cm^−1^) 3391 (OH), 1672 (C=O); ^1^H-NMR (CDCl_3,_ δ ppm, *J* Hz) 7.43 (s, 1H, OH), 7.37 (d, 1H, J=7.1, H-1), 7.25–7.18 (m, 3H, 2 Ar-H and H-5′), 7.13 (t, *J* = 7.3, 1H, Ar-H), 6.94 (s, 1H, H-6), 6.67 (s, 1H, H-8), 6.59 (d, 1H, *J* = 3.4, H-3′), 6.45 (m, 1H, H-4′), 4.86 (sept, *J* = 7.0, 1H, NCHMe_2_), 1.72 (d, 6H, *J* = 7.0, CH_3_); ^13^C-NMR (CDCl_3_, δ ppm) 185.9 (C-10), 155.9 (C-4b), 154.2 (C-2′), 150.0 (C-9), 142.9 (C-5a), 141.7 (C-5′), 140.5 (C-10a), 136.3 (C-7), 132.3 (C-3), 129.3 (C-2), 128.4 (C-4a), 123.5 (C-1), 120.1 (C-4), 116.0 (C-9b), 113.0 (C-8), 104.9 (C-4′), 104.1 (C-3′), 100.3 (C-6), 49.6 (NCHMe_2_), 21.9 (2 CH_3_); MS-ESI (*m/z*) 709.3 [2 M+Na]^+^, 366.2 [M+Na]^+^, 344.2 [M+H]^+^; [M+H]^+^; HRMS-ESI (*m/z*) [M+Na]^+^ calcd for C_22_H_17_NNaO_3_ 366.1094, found 366.1101.

*9-Hydroxy-5,8-diisopropyl-5H-indeno*[1,2-*b*]*indol-10-one* (**6g**)*.* Dark red powder; 20% yield; mp 235 °C; IR (KBr, ν cm^−1^) 3422 (OH), 1665 (C=O); ^1^H-NMR (CDCl_3_ + TFA_,_ δ ppm, *J* Hz) 7.32 (d, 1H, *J* = 7.2, Ar-H), 7.23 (m, 1H, Ar-H), 7.16 (m, 2H, Ar-H), 7.05 (m, 1H, Ar-H), 4.81 (m, 1H, NCHMe_2_), 3.35 (m, 1H, CHMe_2_-8), 1.71 (d, 6H, *J* = 7, 2 CH_3_), 1.26 (d, 6H, *J* = 6.9, 2 CH_3_); ^13^C-NMR (CDCl_3_ + TFA_,_δ ppm) 187.63 (C-10), 158.38 (C-4b), 146.14 (C-5a), 141.24 (C-4a), 140.75 (C-10a), 135.45 (C-9), 132.93 (C-3), 130.36 (C-2), 129.47 (C-8), 124.30 (C-1), 123.49 (C-7 or C-6), 123.38 (C-6 or C-7), 120.85 (C-4), 114.68 (C-9a or C-9b), 113.81 (C-9b or C-9a), 50.41 (NCHMe_2_), 26.42 (CHMe_2_-8), 22.73 (2 CH_3_), 21.60 (2 CH_3_); MS-ESI (*m/z*) 661 [2 M+Na]^+^, 342 [M+Na]^+^, 320 [M+H]^+^; HRMS-ESI (*m/z*) [M+Na]^+^ calcd for C_21_H_21_NNaO_2_ 342.1465, found 342.1460.

#### 3.2.6. General Procedure: Synthesis of Substituted 5*H*-Indeno[1,2-*b*]indole-6,9,10-triones **7a**–**g**

The corresponding 9-hydroxyindeno[1,2-*b*]indolone **6a**–**g** and salcomine (1.8 eq.) were introduced into 5 mL DMF under oxygen and the solution was stirred at room temperature for 27 h. The reaction mixture was then poured into ice water to afford the crude product as a red powder after filtration. The filtrate was extracted with diethyl ether and combined with the precipitate. The crude product **7a**–**g** was finally purified by column chromatography on silica (ethyl acetate/cyclohexane: 1/2).

*5-Isopropyl-7-methyl-5H-indeno*[1,2-*b*]*indole-6,9,10-trione* (**7a**). Red powder; 67% yield; mp 235 °C; IR (KBr, ν cm^−1^) 1721 (C=O), 1660 (C=O), 1647 (C=O); ^1^H-NMR (CDCl_3,_ δ ppm, *J* Hz) 7.62 (d, 1H, *J* = 7.2, H-1), 7.43 (dt, *J* = 0.9 and 7.5 1H, H-4), 7.42 (m, 1H, H-3), 6.50 (q, J = 1.5, 1H, H-8), 5.83 (br s, 1H, NCHMe_2_), 2.10 (d, 3H, *J* = 1.5, CH_3_-7), 1.69 (d, 6H, *J* = 7.1, NCHMe_2_); ^13^C RMN (CDCl_3,_, δ ppm) 183.6 (C-10), 181.8 (C-9), 178.6 (C-6), 154.8 (C-4b), 146.7 (C-5a), 140.2 (C-4a), 134.4 (C-10a), 134.1 (C-9b), 133.3 (C-4), 132.7 (C-8), 130.0 (C-2), 124.7 (C-1), 123.3 (C-9a), 121.6 (C-7), 121.2 (C-3), 50.6 (NCHMe_2_), 21.1 (2CH_3_), 16.4 (CH_3_); MS-ESI (*m/z*) 633.2 [2 M+Na]^+^, 328.1 [M+Na]^+^, 306.1 [M+H]^+^; HRMS-ESI (*m/z*) [M+Na]^+^ calcd for C_19_H_15_NNaO_3_ 328.0944, found 328.0945.

*5,7-Diisopropyl-5H-indeno*[1,2-*b*]*indole-6,9,10-trione* (**7b**). Red powder; 74% yield; mp 230.4 °C; IR (KBr, ν cm^−1^) 1716 (C=O), 1656 (C=O), 1641 (C=O); for complete NMR data see [22]; MS-ESI (*m/z*) 689.7 [2 M+Na]^+^, 356.3 [M+Na]^+^, 334.4 [M+H]^+^; HRMS-ESI (*m/z*) [M+Na]^+^ calcd for C_21_H_19_ NNaO_3_ 356.1257, found 356.1254.

*5-Isopropyl-7-phenyl-5H-indeno*[1,2-*b*]*indole-6,9,10-trione* (**7c**)*.* Red powder; yield 44%; mp 230.4 °C; IR (KBr, ν cm^−1^) 1715 (C=O), 1658 (C=O), 1591 (C=O); ^1^H-NMR (CDCl_3,_ δ ppm, *J* Hz) 7.65 (m, 1H, H-1), 7.50–7.45 (m, 7H, Ar-H), 7.35 (m, 1H, Ar-H), 6.75 (s, 1H, H-8), 5.86 (br s, 1H, NCHMe_2_), 1.72 (d, 6H, *J* = 7.0, 2CH_3_); ^13^C-NMR (CDCl_3,_, δ ppm) 183.8 (C-10), 181.9 (C-9), 177.9 (C-6), 155.5 (C-4b), 147.6 (C-5a), 140.4 (C-4a), 134.6 (C-10a), 134,4 (C-9b), 133.9 (C-1′), 133.6 (C-8), 133.2 (C-4), 130.3 (C-2), 130.1 (C-4′**),** 129.9 (C-3′ et C-5′), 128.7 (C-2′ et C-6′), 125.0 (C-1), 121.9 (C-3, C-9a and C-7), 51.0 (NCHMe_2_), 21.3 (2 CH_3_); MS-ESI (*m/z*) 757.3 [2 M+Na]^+^, 390.2 [M+Na]^+^, 368.1 [M+H]^+^; HRMS-ESI (*m/z*) [M+Na]^+^ calcd for C_24_H_17_NNaO_3_ 390.1101, found 390.1094.

*7-(4-Fluorophenyl)-5-isopropyl-5H-indeno*[1,2-*b*]*indole-6,9,10-trione* (**7d**). Red powder**;** 58% yield; mp 258.1 °C; IR (KBr, ν cm^−1^) 1716 (C=O), 1650 (C=O), 1599 (C=O); for complete NMR data see [22]; MS-ESI (*m/z*) 793.3 [2 M+Na]^+^, 408.1 [M+Na]^+^, 386.2 [M+H]^+^; HRMS-ESI (*m/z*) [M+Na]^+^ calcd for C_24_H_16_FNNaO_3_ 408.1006, found 408.1003.

*7-Furan-2-yl-5-isopropyl-5H-indeno*[1,2-*b*]*indole-6,9,10-trione* (**7e**)*.* Red orange powder; 58% yield; mp 216.1 °C; IR (KBr, ν cm^−1^) 1713 (C=O), 1655 (C=O), 1586 (C=O); ^1^H-NMR (CDCl_3,_ δ ppm, *J* Hz) 7.62 (d, 1H, *J =* 7.2, H-Ar), 7.55 (d, 1H, *J* = 1.7, H-5′), 7.50 (d, 1H, *J* = 3.4, H-3′), 7.43 (m, 2H, H-Ar), 7.30 (m, 1H, H-Ar), 6.98 (s, 1H, H-8), 6.57 (dd, 1H, *J* = 1.8 and 3.5, H-4′), 5.85 (br s, 1H, NCHMe_2_), 1.72 (d, 6H, *J* = 7.1, 2 CH_3_); ^13^C-NMR (CDCl_3_, δ ppm) 183.4 (C-10), 181.4 (C-9), 176.2 (C-6), 155.0 (C-4b), 147.1 (C-5a), 145.0 (C-5′), 140.1 (C-4a), 134.7 (C-10a), 134.2 (C-2′), 133.9 (C-9b), 133.3 (C-4), 129.9 (C-2), 126.1 (C-8), 124.6 (C-1), 123.5 (C-9a), 121.7 (C-3 and C-7), 117.9 (C-4′ or C-3′), 113.2 (C-3′ or C-4′), 50.4 (NCHMe_2_), 21.0 (2 CH_3_); MS-ESI (*m/z*) 737.2 [2 M+Na]^+^, 380.1 [M+Na]^+^, 358.1 [M+H]^+^; HRMS-ESI (*m/z*) [M+Na]^+^ calcd for C_22_H_15_NNaO_4_ 380.0893, found 380.0891.

*5-Isopropyl-8-methyl-5H-indeno*[1,2-*b*]*indole-6,9,10-trione* (**7f**). Red powder; 95% yield; IR (KBr, ν cm^−1^) 1717 (C=O), 1642 (C=O), 1656 (C=O); ^1^H-NMR (CDCl_3_ + TFA_,_ δ ppm, *J* Hz) 7.55 (d, 1H, *J* = 7.1, Ar-H), 7.47–7.40 (m, 2H, 2 Ar-H), 7.30 (m, 1H, Ar-H), 6.51 (s, 1H, H-7), 5.71 (m, 1H, NCHMe_2_), 2.1 (s, 3H, CH_3_-8), 1.68 (d, 6H, *J* = 7.0, NCHMe_2_); ^13^C-NMR (CDCl_3_ + TFA, δ ppm) 185.8 (C-10), 182.4 (C-9), 179.0 (C-6), 155.8 (C-4b), 145.6 (C-5a), 139.6 (C-4a), 134.3 (C-7), 134.1 (C-4), 133.9 (C-9b), 130.4 (C-2), 125.1 (C-1), 122.1 (C-3), 121.6 (C-9a), 50.8 (NCHMe_2_), 20.8 (2CH_3_), 15.2 (CH_3_); MS-ESI (*m/z*) 633.3 [2 M+Na]^+^, 328.1 [M+Na]^+^, 306.2 [M+H]^+^; HRMS-ESI (*m/z*) [M+Na]^+^ calcd for C_21_H_19_NNaO_3_ 328.0944, found 328.0955.

*5,8-Diisopropyl-5H-indeno*[1,2-*b*]*indole-6,9,10-trione* (**7g**)*.* Red powder; 14% yield; mp 226 °C; IR (KBr, ν cm^−1^) 1710 (C=O), 1643 (C=O), 1604 (C=O); ^1^H-NMR (CDCl_3,_ δ ppm, *J* Hz) 7.47 (d, 1H, *J* = 7.0, H-1), 7.30–7.24 (m, 2H, Ar-H), 7.15 (m, 1H, Ar-H), 6.22 (s, 1H, H-7), 5.64 (br s, 1H, NCHMe_2_), 2.99 (sept, 1H, *J* = 6.7, CHMe_2_-8), 1.53 (d, 6H, *J* = 7.0, 2 CH_3_), 1.01 (d, 6H, *J* = 6.8, 2 CH_3_); ^13^C-NMR (CDCl_3_, δ ppm) 183.6 (C-10), 181.3 (C-9), 179.0 (C-6), 154.5 (C-4b), 154.1 (C-5a), 139.9 (C-4a), 134.1 (C-10a), 133.5 (C-9b), 133.2 (C-4), 131.7 (C-7), 129.8 (C-2), 124.5 (C-1), 123.5 (C-9a), 121.3 (C-3), 50.1 (NCHMe_2_), 26.5 (CHMe_2_-8), 21.7 (2 CH_3_), 20.9 (2 CH_3_); MS-ESI (*m/z*) 689.3 [2 M+Na]^+^, 356.2 [M+Na]^+^, 334.2 [M+H]^+^; HRMS-ESI (*m/z*) [M+Na]^+^ calcd for C_21_H_19_NNaO_3_ 356.1257, found 356.1253.

### 3.3. Biological Activities

#### 3.3.1. Preparation of Recombinant Human CK2 Holoenzyme

The preparation of the human recombinant CK2 holoenzyme was performed according to a protocol described previously [29]. For the expression of the α-subunit (CSNK2A1) and β-subunit (CSNK2B) of the human protein kinase CK2 a pT7-7 expression system in *Escherichia coli* BL21(DE3) was used. Newly transformed starter cultures were grown overnight at 37 °C in LB-medium to the stationary phase. New medium was inoculated with the separate starter cultures for both subunits and cultivated until an OD_500_ of 0.6 was reached. Expression was induced by addition of IPTG (1 mM final concentration) and carried out at 30 °C for 5–6 h for CSNK2A1 and at 37 °C for 3 h for CSNK2B. Cells were harvested by centrifugation (6000× *g* for 10 minutes at 4 °C) and disrupted by sonication (three times 30 seconds on ice). Preparations were then centrifuged to remove the cells debris and the bacterial extracts for both subunits were combined and purified by a three-column procedure. Fractions exhibiting CK2 activity were combined and analyzed by SDS-PAGE and Western Blot.

#### 3.3.2. Capillary Electrophoresis Based Assay for the Testing of Inhibitors of the Human CK2

Testing of the inhibitors of the human CK2 was performed by the recently established capillary electrophoresis based CK2 activity assay of Gratz et al. [23]. Therefore, 2 µL of the dissolved inhibitors (stock solution in DMSO) were mixed with 78 µL of CK2 supplemented kinase buffer which was composed of 1 µg CK2 holoenzyme, 50 mM Tris/HCl (pH 7.5), 100 mM NaCl, 10 mM MgCl_2_ and 1 mM DTT. The reaction was initiated by the addition of 120 µL assay buffer, which was composed of 25 mM Tris/HCl (pH 8.5), 150 mM NaCl, 5 mM MgCl_2_, 1 mM DTT, 100 µM ATP and 0.19 mM of the substrate peptide RRRDDDSDDD. The reaction was carried out for 15 minutes at 37 °C and stopped by the addition of 4 µL EDTA (0.5 M). Subsequently the reaction mixture was analyzed by a PA800 capillary electrophoresis from Beckman Coulter (Krefeld, Germany). Acetic acid (2 M, adjusted with conc. HCl to a pH of 2.0) was used as the electrolyte for electrophoretic separation. The separated substrate and product peptide were detected at 214 nm using a DAD-detector. Pure solvent was used as negative control (0% inhibition), assays devoid of CK2 were used as positive control (100% inhibition). For primary testing an inhibitor concentration of 10 µM was used. Compounds that revealed at least 50% inhibition at 10 µM were used for IC_50_ determinations. For the determination of IC_50_, inhibition was determined using nine inhibitor concentrations ranging from 0.001 µM to 100 µM. IC_50_ were calculated from the resulting dose-response curves.

#### 3.3.3. Mode of Inhibition and Determination of K_i_

The CE based CK2 activity assay as described was used to investigate whether **5a** and **7a** inhibit CK2 by an ATP competitive mode. For this purpose, enzymatic reactions and IC_50_ determinations were performed as described in Section 3.3.2., but varying compositions of the assay buffer with respect to the ATP concentration were used. IC_50_ values were measured at six different final ATP concentrations ranging from 6 to 600 µM. The obtained IC_50_ values were subsequently used to determine the K_i_ values of **5a** and **7a**. For this purpose, reaction velocities at different inhibitor concentrations were plotted in a Lineweaver-Burk diagram against the varied ATP concentrations and the corresponding $-1/K_{m}^{app}$ were obtained by the intercepts with the abscissa. Finally the $K_{m}^{app}$ values as obtained were plotted against the different inhibitor concentrations. By this method, the K_i_ values with a negative sign were given by the intercepts of the linear slope with the abscissa.

#### 3.3.4. Cytotoxicity Assay on Cell Lines

All cells were grown in DMEM medium supplemented with 10% fetal calf serum, glutamine (2 mM), penicillin (100 U/mL), and streptomycin (50 μg/mL) at 37 °C under a humidified 5% CO_2_ atmosphere (Gibco). The assays were performed in sterile 96-wells microtiter plates (Falcon). Cells were plated at 500 (NIH-3T3), 750 (WI-38) or 1000 (MEF; HEK293T) cells/100 µL/well. Drug solutions (10 μL per well) of appropriate concentration (prepared by dilution in the culture medium from 2 mM stock acetonitrile solution) were added 24 h after cell seeding. For ellagic acid and emodin, DMSO was used to prepare the stock solution. After 96 h, the cytotoxicity is evaluated using the Cell Proliferation Reagent WST-1 following the procedure described by the manufacturer (Roche Applied Science). The drugs were assayed at 10, 5, 1, 0.2 and 0.04 µM in triplicate. The vehicle was used as control. The experiments were repeated three times.

For each compound tested, the EC_50_ (concentration of drug needed to inhibit cell growth/viability by 50%) was generated from the dose-response curves for each cell line.

### 3.4. Crystallography

The structure of compound **4d’** has been established by X-ray crystallography (Figure 4). Yellow single crystal (0.76 × 0.13 × 0.09 mm^3^) of **4d’** was obtained after 10 days at 17 °C by slow evaporation from a methanol/chloroform (80/20) solution. It crystallized in the monoclinic space group P21/c, *a* = 13.103(2) Å, *b* = 10.0799(11) Å, *c* = 16.411(2) Å, α = 90°, β = 95.439(13)°, γ = 90°, *V* = 2157.8(5) Å^3^, Z = 4, δ(calcd) = 1.310 Mg.m^−3^, FW = 425.44 for C_24_H_24_FNO_5_, *F*(000) = 896, final R indexes were *R*_1_ = 0.0519 (I > 2σ(I)) and *wR*_2_ = 0.1437 (all data). Crystallographic data were acquired on a Bruker-Nonius Kappa CCD. Full crystallographic results have been deposited at the Cambridge Crystallographic Data Centre (CCDC-977015), UK, as supplementary Material [30]. The data were corrected for Lorentz and polarization effects and for empirical absorption correction [31]. The structure was solved by direct methods with ShelxS 2013 [31] and refined using ShelxL 2013 programs [32], both pieces of software were found within the OLEX2 package [21].

### 3.5. Molecular Modeling

All four inhibitors **5a**, **7a**, **5h** [13], and **7h** [14] were structurally aligned using the program vROCS [25]. For this a pharmacophore match based on shape and H-bond donor and acceptor groups with the co-crystallized inhibitor (from PDB code: 3OWJ) was performed. Subsequently, the inhibitors were energy minimized in the binding region of CK2 (from PDB code: 3OWJ) using the program Moloc [26].

## 4. Conclusions

A library of twenty-one new indeno[1,2-*b*]indole derivatives was synthesized and evaluated as inhibitors of human protein kinase CK2. This preliminary SAR study confirmed that compounds with substituents at position 5 (C-ring) and position 7 (D-ring) of the indeno[1,2-*b*]indole scaffold showed CK2 inhibitory activity at micromolar and submicromolar range of concentrations (e.g., compounds 5a, 5b and 7a have IC_50_ values of 0.17, 0.61 and 0.43 µM, respectively). In comparison with three standards, we showed that compound 5b and 7a are as active as emodin but less potent than ellagic acid and TBB. However compound 5a is a better CK2 inhibitor than emodin (0.17 *versus* 0.58 µM). Furthermore, the low cytotoxicity of compound 5a against four cell lines encourages us to continue our medicinal chemistry investigations. For example 5,6,7,8-tetrahydroindeno[1,2-*b*]indole-9,10-diones 5, 9-hydroxy-5*H*-indeno[1,2-*b*]indol-10-ones 6 and 5*H*-indeno[1,2-*b*]indole-6,9,10-triones 7 were included in building a 3D-QSAR model. The third generation of indeno[1,2-*b*]indoles as CK2 inhibitors are actually synthesized and evaluated.

## Supplementary Files

Supplementary File 1
