# Supplementary material for: Synthesis, Biological Evaluation and Molecular Modeling of Substituted Indeno[1,2-b]indoles as Inhibitors of Human Protein Kinase CK2
Source: Pharmaceuticals (Basel). 2015 Jun 8;8(2):279–302. doi: 10.3390/ph8020279 (PMC4491662; doi:10.3390/ph8020279)
Supplement: Supplementary File 1 [file pharmaceuticals-08-00279-s001.doc]

Supplementary Materials


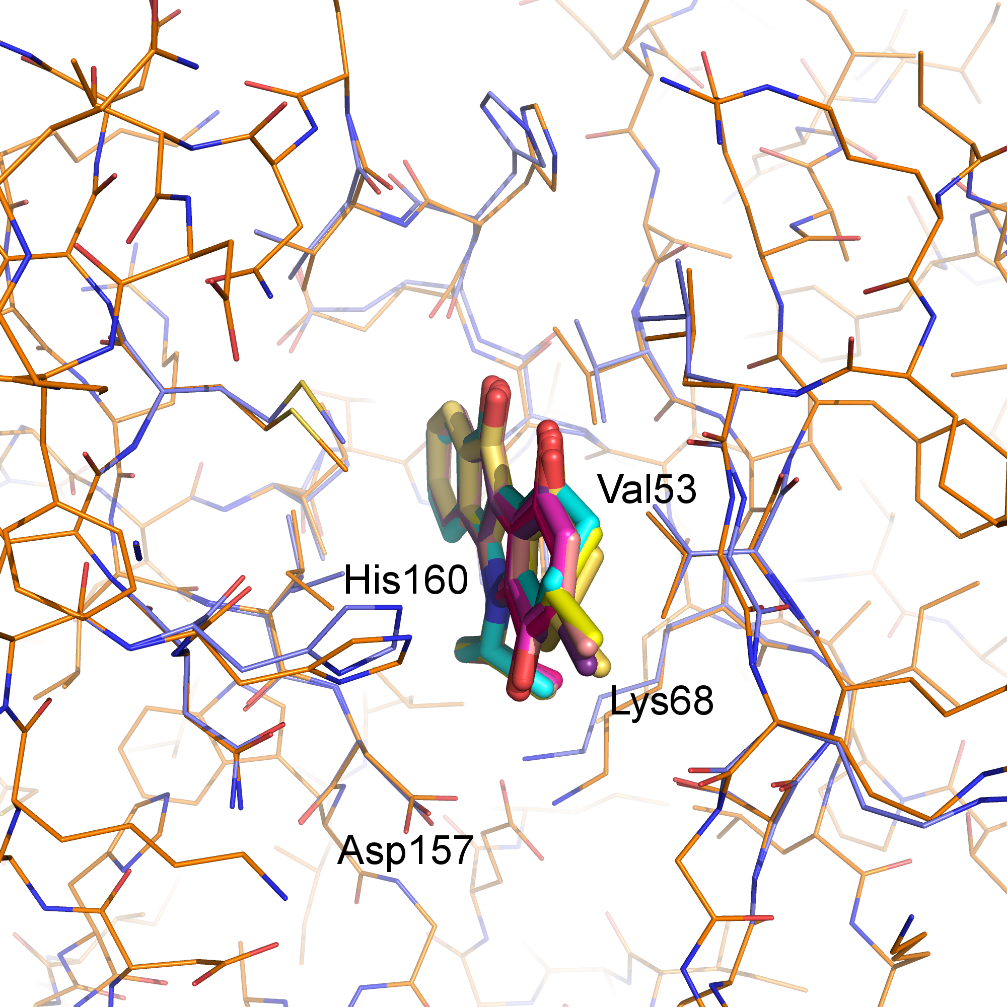


**Figure S1.** Overlay of the crystal structure (blue) of CK2 from PDB ID 3OWJ [1] and the structure obtained by energy minimization with Moloc in the presence of **7h**, allowing all residues within 5 Å of the ligand to move (orange). Only minor differences are observed between the two protein structures. In addition, ligands **5a**, **7a**, **5h**, and **7h** are depicted in stick representation after energy minimization with and without mobile binding site residues. The binding modes of each ligand are highly similar irrespective of the treatment of the binding site residues.


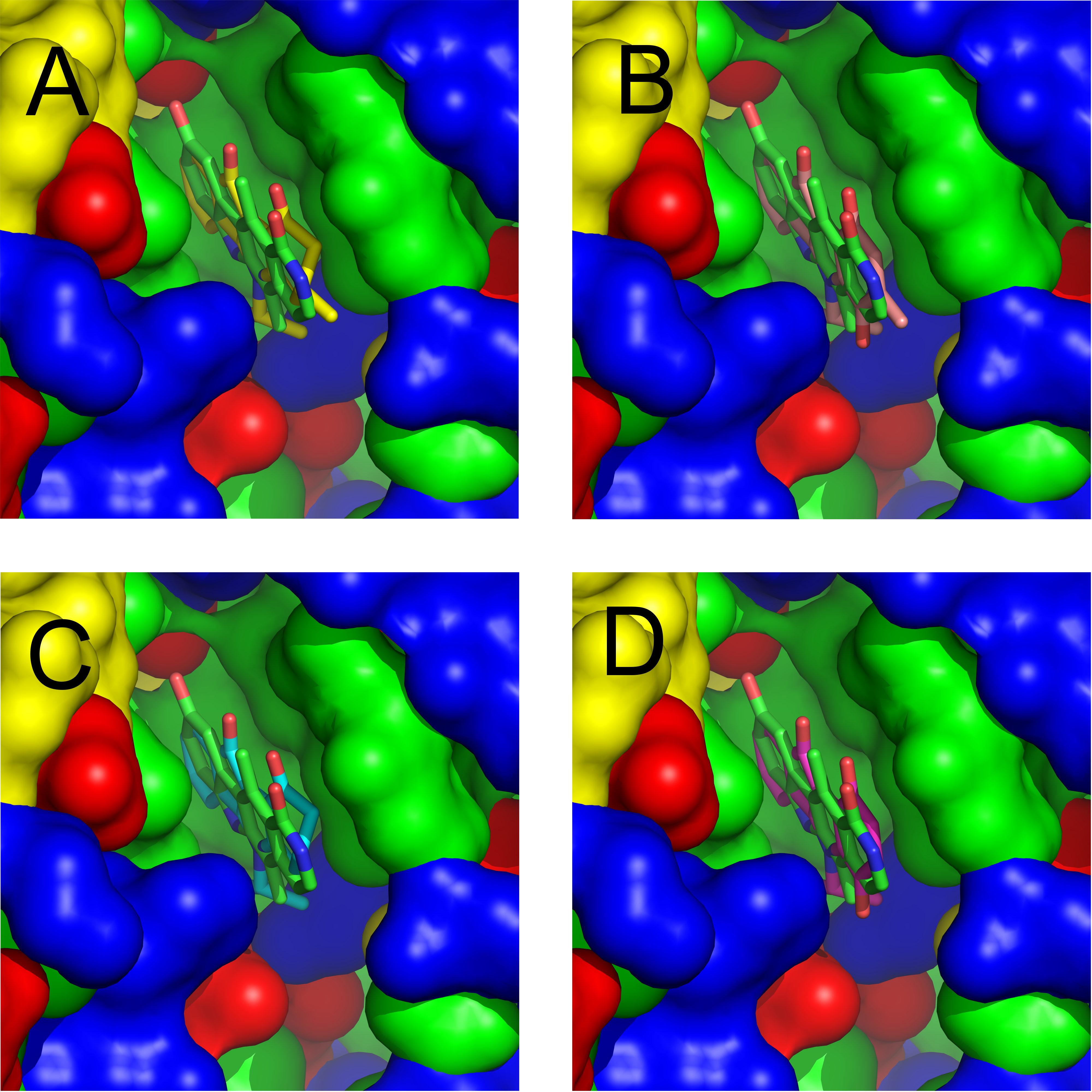


**Figure S2.** Overlay of the inhibitor 9-hydroxy-5,11-dimethyl-4,6-dihydro-1*H*-
pyrido[4,3-*b*]carbazol-1-one co-crystalized (green) with CK2 (PDB code: 3OWJ) and **A**: **5a**, **B**: **7a**, **C**: **5h** [2], and **D**: **7h** [3] obtained by a structural alignment with the program
vROCS [4] and subsequent energy minimization with the program Moloc [5] in the presence of the enzyme (green: hydrophobic surface patches, yellow: hydrophilic surface patches, blue (red): positively (negatively) polarized surface patches.

**Table S1.** Additional Compounds Used for Bioevaluation.

| **Cpd** | **Structure** | **CK2** | |
| --- | --- | --- | --- |
|  |  | **% inh. 10 μM** | **IC_50_ (µM)** |
| 5h |  | 99 | 0.36 |
| 7h |  | 60 | 5.55 |

**References**

1. Prudent, R.; Moucadel, V.; Nguyen, C.H.; Barette, C.; Schmidt, F.; Florent, J.C.; Lafanechère, L.; Sautel, C.F.; Duchemin-Pelletier, E.; Spreux, E.; *et al*. Antitumor activity of pyridocarbazole and benzopyridoindole derivatives that inhibit protein kinase CK2. *Cancer Res.* **2010**, *70*, 9865–9874.
2. Hundsdörfer, C.; Hemmerling, H.-J.; Götz, C.; Totzke, F.; Bednarski, P.; Le Borgne, M.; Jose, J. Indeno[1,2-*b*]indole derivatives as a novel class of potent human protein kinase CK2 inhibitors. *Bioorg. Med. Chem.* **2012**, *20*, 2282–2289.
3. Hundsdörfer, C.; Hemmerling, H.-J.; Hamberger, J.; Le Borgne, M.; Bednarski, P.; Götz, C.; Totzke, F.; Jose J. Novel indeno[1,2-*b*]indoloquinones as inhibitors of the human protein kinase CK2 with antiproliferative activity towards a broad panel of cancer cell lines. *Biochem. Biophys. Res. Commun.* **2012**, *424*, 71–75.
4. Openeye-Scientific-Software. vROCS. 3.1.1 ed., Openeye Software, Santa Fee, New Mexico, 2011.
5. Muller, K.; Ammann, H.J.; Doran, D.M.; Gerber, P.R.; Gubernator, K.; Schrepfer, G. MOLOC: A molecular modeling program. *Bull. Soc. Chim. Belg.* **1988**, *97*, 655–667.

© 2015 by the authors; licensee MDPI, Basel, Switzerland. This article is an open access article distributed under the terms and conditions of the Creative Commons Attribution license (http://creativecommons.org/licenses/by/4.0/).
